# Synthesis of carbazole and naphthyl-pyrimidine-based inhibitors of *Xanthomonas *for tomato bacterial spot management

**DOI:** 10.1007/s11030-026-11516-3

**Published:** 2026-04-04

**Authors:** Rajeev Shrestha, Rahul Khupse, Gireesh Rajashekara

**Affiliations:** 1 Department of Pathobiology, College of Veterinary Medicine, University of Illinois Urbana- Champaign, Urbana, IL 61802 USA; 2 College of Pharmacy, University of Findlay, Findlay, OH 45840 USA

**Keywords:** Bacterial spot of tomato (BST), *Xanthomonas hortorum* pv. *gardneri* (*Xhg*), Small-molecule growth inhibitors, Structural expansion, small molecule synthesis, Antimicrobial activity

## Abstract

Bacterial spot of tomato (BST), caused by *Xanthomonas* species, is a devastating disease in tomato production, leading to severe yield losses and reduced crop quality. Current control strategies, including cultural management and chemical applications, are increasingly challenged by the emergence of copper- and antibiotic-resistant strains. To address this issue, we are exploring the development of novel small molecule antibacterial as a promising alternative control strategy. Through structural expansion and modification of previously identified anti-*Xanthomonas* leads (**X2**, **X5**, and **X12**), we synthesized a library of small molecules featuring key pharmacophores, including pyrimidine, oxazole, and imidazole. The synthesized compounds were evaluated for their inhibitory activity against *Xanthomonas hortorum* pv. *gardneri* (*Xhg*). Three derivative 4-(2-(1-(2-hydroxy-3-((2-(naphthalen-1-yl)pyrimidin-5-yl)methoxy)propyl)piperidin-2-yl)ethyl)phenol (**X2-c**), 4-(2-(1-(3-(9*H*-carbazol-9-yl)-2-hydroxypropyl)piperidin-2-yl)ethyl)phenol (**X12**-**k**), and 1-(9*H*-carbazol-9-yl)-3-(2-(2-(pyridin-2-yl)ethyl)piperidin-1-yl)propan-2-ol (**X12**-**l**) demonstrated promising antibacterial effects, exhibiting minimum inhibitory concentrations (MIC) of 25–50 µM and minimum bactericidal concentrations (MBC) of 50–100 µM. **X12-l** exhibited rapid bactericidal activity against *Xhg* within 1 h, whereas **X2-c** and **X12-k** required 4 h and 6 h, respectively, in a time–kill assay. Furthermore, in silico predictions for compounds **X2-c**, **X12-k**, and **X12-l** indicate an optimal balance of molecular size, polarity, and lipophilicity, conforming to Lipinski’s rule and exhibiting acceptable absorption, distribution, metabolism, and excretion (ADME) profiles. These small molecules can be potentially developed into a useful tool for controlling *Xanthomonas* in tomato production. Future studies will focus on elucidating their mode of action and evaluating their safety and efficacy in seeds and seedlings.

**Supplementary Information:**

The online version contains supplementary material available at 10.1007/s11030-026-11516-3.

## Introduction

Over the last few decades, bacterial spot of tomato (BST) has emerged as a devastating tomato disease, mainly elicited by *Xanthomonas* species, including *X. hortorum* pv. *gardneri* (*Xhg*), *X. perforans*, *X. vesicatoria*, and *X. euvesicatoria* [1, 2]. According to the 2016 USDA National Institute of Food and Agriculture report, BST caused an estimated $7–8 million in losses in the midwestern U.S. in 2010 [3]. These highly virulent pathogens thrive in warm and humid conditions, leading to rapid spread and persistent problems in field and greenhouse tomato cultivation [4]. The pathogens infect fruits, leaves, and stems, causing necrotic lesions, defoliation, impaired photosynthesis, and fruit blemishes, severely reducing crop quality and marketability [5, 6]. BST substantially threatens global tomato production, with yield losses as high as 50% in severely infested regions [3]. To ensure food security and the integrity of tomato agricultural systems, early management of BST is a significant factor [7–9].

Effective management of bacterial spot of tomato remains challenging due to the highly resistant nature of all *Xanthomonas spp*. associated with the disease, the seed-borne infection of the pathogen, its rapid dissemination under optimal environmental conditions, and the presence of copper- and streptomycin-resistant strains [10]. Current strategies primarily rely on integrated cultural practices and chemical treatments [10–12]. Cultural measures include crop rotation, using resistant seed varieties, and strict sanitation to eliminate infected plant debris [13]. These practices, however, can be limited by the availability of non-host crops and the challenge of managing water and temperature conditions [14]. Copper-based bactericides, such as copper hydroxide and copper oxychloride, are commonly used as preventive sprays, but their effectiveness is often reduced by the emergence of resistant bacteria [5, 15, 16]. In addition, antibiotics such as streptomycin, oxytetracycline, and kasugamycin have been used; however, their use is increasingly restricted in farm practices due to regulatory concerns and the rise of antibiotic-resistant strains of *Xanthomonas* spp [17, 18]. In light of the growing resistance to both antibiotics and copper, there is a pressing need to develop alternative control strategies. Therefore, developing novel small-molecule inhibitors is an effective strategy for combating antibiotic resistance and enabling sustainable crop disease management.

Many small molecule antimicrobials bearing phenolic or heterocyclic groups play a vital role in combating bacterial infections [19]. The molecular structure of these antimicrobials enables effective penetration of the protective layers of bacterial cells that infect plants, such as biofilms and outer membranes. Upon cellular entry, they can disrupt the essential bacterial processes, including cell wall synthesis, protein production, and DNA replication, ultimately inhibiting bacterial growth and protecting the plant [20, 21]. Recent studies demonstrated that new strategies employing small-molecule inhibitors are promising for controlling bacterial diseases in tomato caused by *Xanthomonas* spp [15, 22–27]. The small molecules, including 3-indolyacetonitrile [22], *N*-acetylcysteine [23], piperidine, pyrrolidine [24], carvacrol [15], coumarin [25], uniconazole [26], and salicylic acid [27], have demonstrated activity against *Xanthomonas* spp. in tomato. In this vein, as part of our continuous efforts to discover novel small-molecule inhibitors, we successfully conducted high-throughput screening of over 4,182 compounds. We identified a variety of phenolic and heterocyclic compound leads, including benzimidazoles, imidazoles, quinolines, acrylidines, carbazoles, guanidines, piperidines, pyrrolidones, and phenol derivatives that exhibited inhibitory activity against *Clavibacter michiganensis subsp. Michiganensis* [28], *Pseudomonas syringae* [29], *Erwinia tracheiphila* [30], *Acidovorax citrulli* [31], and *Xanthomonas* spp [32]. With their demonstrated bioactivity and structural properties, these lead molecules offer a valuable starting point for the design of new classes of antibiotics.

**Fig. 1:**
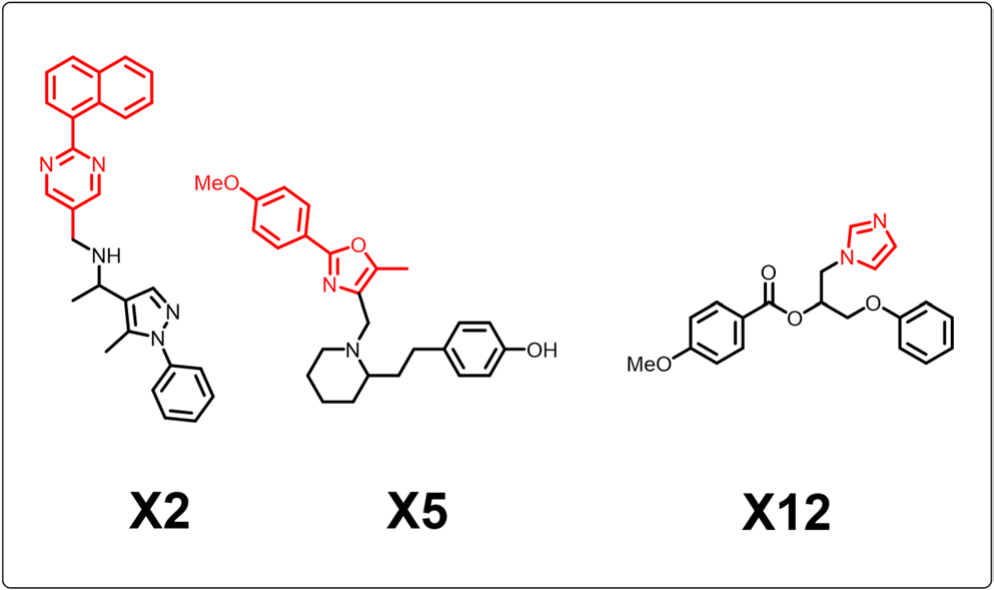
Structures of previously reported lead small molecules [32]

The structural expansion of lead compounds is a well-established drug-design strategy used to modify the core scaffolds and generate novel molecular frameworks, enabling the discovery of new bioactive hits with innovative core architectures for alternative antibiotic development [33, 34]. Based on initial screening of the library of compounds, we identified three best compounds: 1-(5-methyl-1-phenyl-1*H*-pyrazol-4-yl)-*N*-((2-(naphthalen-1-yl)pyrimidin-5-yl)methyl)ethan-1-amine (**X2**), 4-(2-(1-((2-(4-methoxyphenyl)-5-methyloxazol-4-yl)methyl)piperidin-2-yl)ethyl)phenol (**X5**), and 1-(1*H*-imidazol-1-yl)-3-phenoxypropan-2-yl 4-methoxybenzoate (**X12**) that demonstrated the promising inhibitory activity against *Xanthomonas* spp. (Fig. 1) [32]. These compounds also exhibited bactericidal activity against biofilm-embedded *Xanthomonas* and showed no observable phytotoxic effects on tomato seedlings even at 500 µM. Moreover, application of these compounds at 500 µM significantly suppressed disease development in *Xhg*-infected tomato plants under both preventive and curative treatments. In addition, the compounds also effectively reduced *Xanthomonas* populations in artificially infested tomato seeds at 200 µM [32]. To further enhance antibacterial efficacy against *Xanthomonas* spp., we pursued structural expansion and derivatization of the lead compounds **X2**, **X5**, and **X12**, aiming to improve antibacterial potency while retaining their core bioactive scaffolds, including 2-(naphthalen-1-yl)pyrimidine, oxazole, and imidazole. Notably, the key pharmacophores, including 2-(naphthalen-1-yl)pyrimidine, oxazole, and imidazole are core heterocyclic motifs found in several established antibiotics and are well recognized for their broad medicinal relevance. These pyrimidine-, oxazole-, and imidazole-based scaffolds provide a strong foundation for the rational design of novel antibacterial compounds through strategic linkage with other bioactive groups [35–37]. Furthermore, carbazole derivatives, characterized by a central pyrrole ring fused between two benzene rings, have emerged as potent antimicrobial agents against clinical pathogens such as *Staphylococcus aureus*, including Methicillin-resistant *S. aureus* (MRSA), *Streptococcus pyogenes*, *Escherichia coli*, *Pseudomonas aeruginosa*, and *Bacillus subtilis* [38–40]. The recent study highlighted that a prenylated carbazole derivative demonstrated antimicrobial activity against plant pathogens, notably *Xanthomonas oryzae pv. oryzae* (*Xoo*) with MIC value of 1.56 µg/mL [41]. Building on these findings, we strategically replaced the imidazole moiety with a carbazole unit in new **X12** derivatives. This modification was designed to expand the aromatic framework and enhance π-stacking interactions with bacterial targets, potentially improving potency [38]. Meanwhile, the isopropanolamine unit, characterized by a *β*-amino alcohol structure, has been extensively employed in antimicrobial drug design due to its ability to enhance membrane permeability and hydrogen bond formation, thereby improving overall biological performance [42–44]. Notably, isopropanolamine derivatives have demonstrated excellent bioactivity against the destructive plant pathogens, *Xoo* and *Xanthomonas axonopodis* pv. *citri*, with EC_50_ values of 0.34 and 2.1 µg/mL respectively [45]. Thus, the rational design of imidazole, pyrimidine, or carbazole derivatives decorated with an isopropanolamine moiety may represent a promising strategy for developing effective antibacterial agents against *Xanthomonas* spp.

Here, we describe the structural expansions of leads **X2**, **X5**, and **X12** to generate new compounds with better activity against *Xanthomonas* spp. These compounds were synthesized in multiple steps to combine the scaffolds from the original active hits. These molecules were screened against *Xhg* to determine their minimum inhibitory concentration (MIC) and minimum bactericidal concentration (MBC). Through this screening, we identified three improved analogs- 4-(2-(1-(2-hydroxy-3-((2-(naphthalen-1-yl)pyrimidin-5-yl)methoxy)propyl)piperidin-2-yl)ethyl)phenol (**X2-c)**, 4-(2-(1-(3-(9*H*-carbazol-9-yl)-2-hydroxypropyl)piperidin-2-yl)ethyl)phenol **(X12-k)**, and 1-(9*H*-carbazol-9-yl)-3-(2-(2-(pyridin-2-yl)ethyl)piperidin-1-yl)propan-2-ol **(X12-l)** with MIC values ranging from 25 to 50 µM and MBC values between 50 and 100 µM. These compounds show significant potential for controlling *Xanthomonas* species. Furthermore, we evaluated the drug-likeness of the lead compounds **X2-c**, **X12-k**, and **X12-l** using in silico pharmacological and ADME predictions conducted with SwissADME and pkCSM pharmacokinetic tools.

## Result and discussion

### Chemistry

**Fig. 2:**
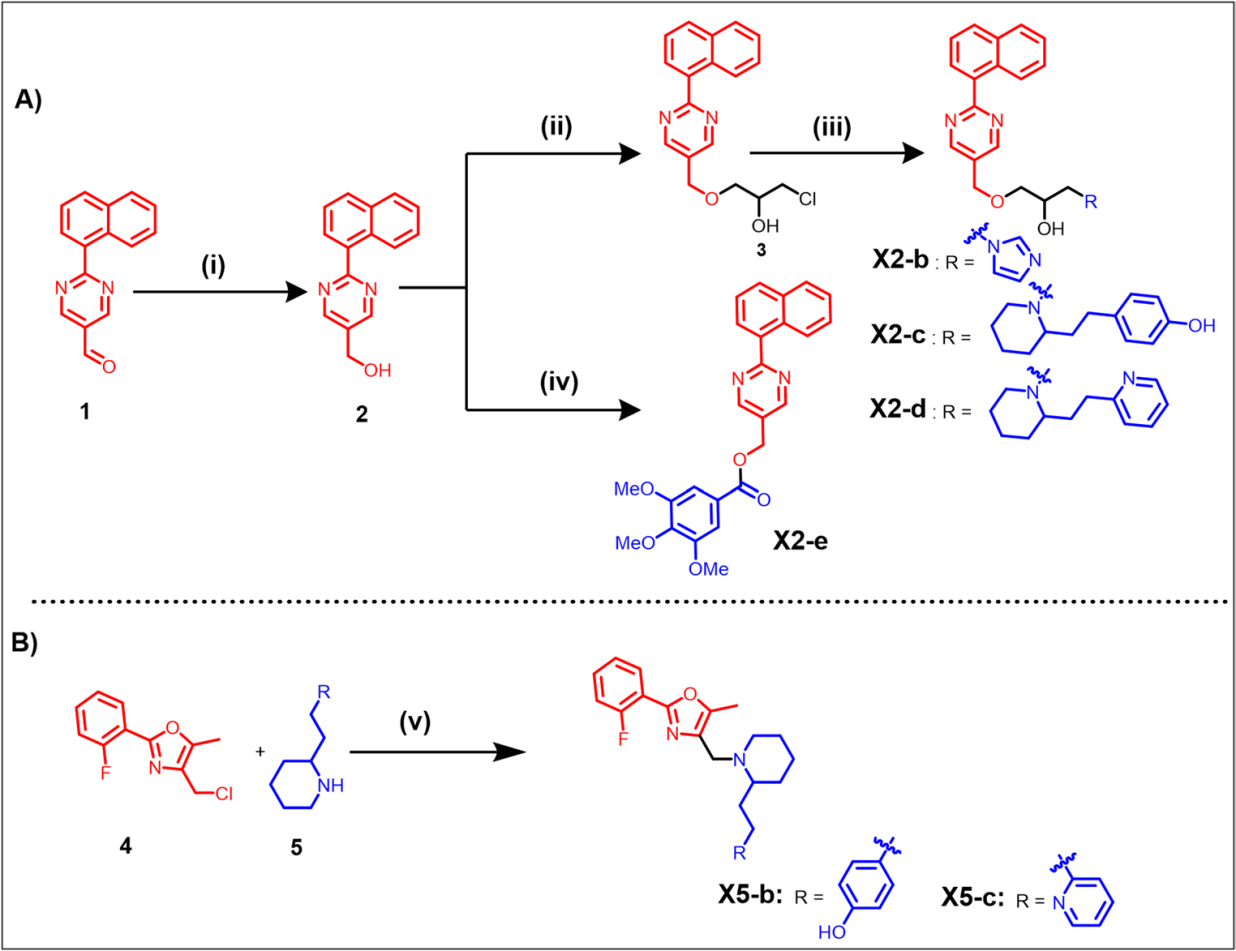
Synthesis route of **A**) **X2** (**X2-b **to **X2-e**) and **B**) **X5** (**X5-b** and **X5-c**) derivatives. *Reagents and conditions*: (i) NaBH_4_, MeOH, 0 °C to rt; (ii) epichlorohydrin, KOH , ACN, 60 °C; (iii) imidazole or 4-(2-(piperidin-2-yl)ethyl)phenol or 2-(2-(piperidin-2-yl)ethyl)pyridine, K_2_CO_3_, ACN, 80 °C; (iv) 3,4,5-trimethoxybenzoyl chloride, KOH, EtOH, 80 °C; (v) 4-(2-(piperidin-2-yl)ethyl)phenol or 2-(2-(piperidin-2-yl)ethyl)pyridine, K_2_CO_3_, ACN, 80 °C

**Fig. 3:**
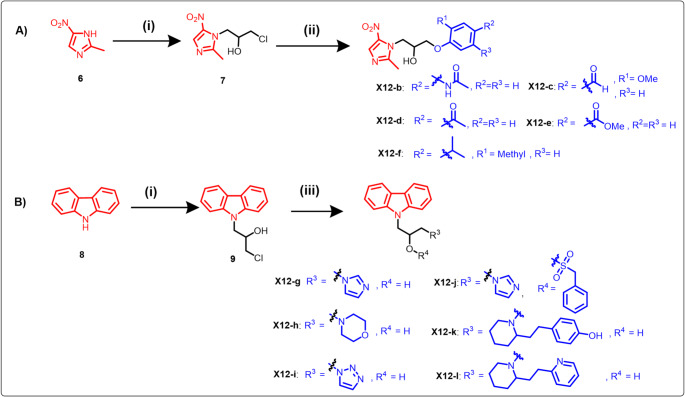
Synthesis route of **X12** derivatives: **A**) **X12-b** to **X12-f** and **B**) **X12-g **to **X12-l**. *Reagents and conditions*: (i) epichlorohydrin, KOH, DMF, 60 °C; (ii) *N*-(4-hydroxyphenyl)acetamide or 4-hydroxy-3-methoxybenzaldehyde or 1-(4-hydroxyphenyl)ethan-1-one or methyl 4-hydroxybenzoate or 4-isopropyl-2-methylphenol, K_2_CO_3_, ACN, 80 °C; (iii) imidazole, morpholine, triazole, phenylmethanesulfonyl chloride, 4-(2-(piperidin-2-yl)ethyl)phenol or 2-(2-(piperidin-2-yl)ethyl)pyridine , K_2_CO_3_ , ACN, 80 °C

A series of novel analogues of **X2**,** X5**, and **X12** were designed by varying the heterocycles and synthesized as depicted in Figs. 2 and 3. The synthesis of compounds **X2-b-X2-e** was initiated from the commercially available 2-(naphthalen-1-yl)pyrimidine-5-carbaldehyde (**1**), following a modified procedure [46, 47] (Fig. 2A). Initially, the starting material **1** was reduced using sodium borohydride to yield (2-(naphthalen-1-yl)pyrimidin-5-yl)methanol (**2**). Afterward, intermediate **2** was reacted with epichlorohydrin under basic conditions in acetonitrile, undergoing nucleophilic ring opening to afford intermediate **3.** Subsequently, the compounds **X2-b** to **X2-d** were synthesized via nucleophilic substitution reactions with the intermediate **3** and either imidazole, 4-(2-(piperidin-2-yl)ethyl)phenol, or 2-(2-(piperidin-2-yl)ethyl)pyridine in the presence of potassium carbonate. Similarly, the compound **X2-e** bearing the ester moiety was synthesized via the direct Schotten-Baumann reaction between intermediate **2** and 3,4,5-trimethoxybenzoyl chloride under alkaline conditions. Furthermore, the compounds **X5-b** and **X5-c** were produced via an S_N_2 nucleophilic substitution reaction between 4-(chloromethyl)-2-(2-fluorophenyl)-5-methyloxazole (**4**) and 4-(2-(piperidin-2-yl)ethyl)phenol or 2-(2-(piperidin-2-yl)ethyl)pyridine (Fig. 2B).

Moreover, the Epoxide-Amine coupling reaction of the epichlorohydrin and 2-methyl-5-nitro-1*H*-imidazole (**6**) or carbazole (**8**) was conducted under basic conditions to achieve intermediates 1-chloro-3-(2-methyl-5-nitro-1*H*-imidazol-1-yl)propan-2-ol (**7**) or 1-(9*H*-carbazol-9-yl)-3-chloropropan-2-ol (**9**) (Fig. 3). The intermediate **7** was reacted with various phenolic derivatives, including *N*-(4-hydroxyphenyl)acetamide or 4-hydroxy-3- methoxybenzaldehyde or 1-(4-hydroxyphenyl)ethan-1-one or methyl 4-hydroxybenzoate or 4-isopropyl-2-methylphenol, to afford the product **X12-b** to **X12-f **(Fig. 3A).

In addition, the intermediate **9** was treated with various *N-*heterocycles such as imidazole, morpholine, or triazole to yield **X12-g** to **X12-i (**Fig. 3B). The compound **X12**-**j** was prepared through the subsequent reaction of **X12**-**g** with phenylmethanesulfonyl chloride in basic media. Moreover, the compounds **X12**-**k** and **X12**-**l** were also synthesized though S_N_2 nucleophilic substitution reaction with **9** and 4-(2-(piperidin-2-yl)ethyl)phenol or 2-(2-(piperidin-2-yl)ethyl)pyridine under basic condition. All molecules were characterized using ^1^H NMR and mass spectrometry. The molecular masses of the synthesized molecules ranged from 291 to 497 Daltons (Supplementary Information). The NMR spectral data are provided in the Supplementary Information.

### Antimicrobial inhibition study

The synthesized compounds were evaluated for their growth inhibitory activity against *Xhg* SM775-12 at concentrations of 25, 50, 100, or 200 µM, and the activities were compared with those of commercially obtained compounds **(X2**,** X2-a**,** X5**,** X5-a**,** X12**, and **X12-a**) (Table 1). Among the synthesized derivatives, **X12-k** displayed the best activity, with 100% *Xhg* growth inhibition at 25 µM, which is superior to all other compounds tested. Similarly, **X2-c** and **X12-l** exhibited complete inhibition at 50 µM, while **X2-d** was required at 100 µM for 98% inhibition. On the other hand, the starting lead compounds **X5** and **X12** demonstrated complete inhibition at 100 µM, whereas **X2** inhibited the bacteria at 200 µM. Notably, **X12-k** displayed eightfold greater potency than **X2** and fourfold greater activity than **X5** and **X12**, confirming its superior antibacterial efficacy.

**Fig. 4:**
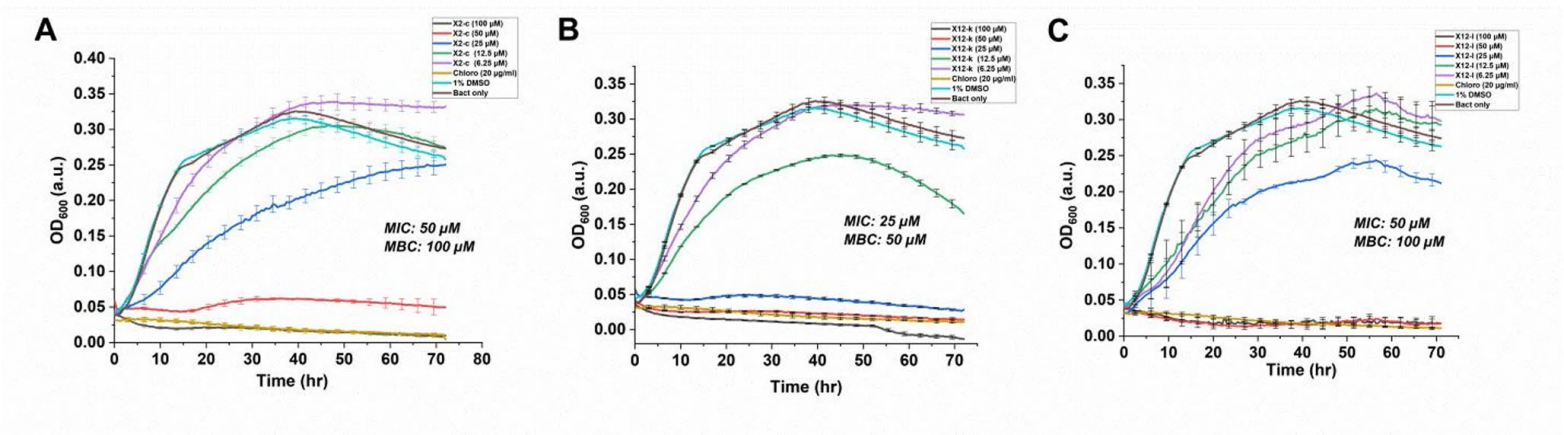
The MIC and MBC of lead compounds **A**) **X2-c**,** B**) **X12-k**, and **C**) **X12-l**. Test compounds were added at different concentrations (6.25–100 µM) in a 96-well plate containing 100 µl of 5 × 10^7^ CFU/ml of *Xhg* SM775-12 and incubated at 28 °C for 72 h in a Tecan Sunrise Kinetic reader. Plates were agitated every 15 min for 30 s at 180 rpm and OD_600_ was measured every 30 min. Bacterial turbidity initially increased and subsequently decreased over time following compound treatment, as monitored by OD_600_ measurements. MIC is the lowest concentration of a compound that inhibits visible bacterial growth (potency), whereas MBC is the lowest concentration that results in bacterial death (efficacy), demonstrated by the absence of colony formation when the treated culture is transferred to agar plates. The MBC was determined by sub-culturing the compound-treated bacterial suspensions from clear (non-turbid) MIC wells onto YDC agar plates and was confirmed by the absence of viable colony formation. Data are presented as mean ± SD from three independent experiments

Following the initial screening, the lead compounds **X2-c**, **X12-k**, and **X12-l** were further evaluated for their minimum inhibitory concentration (MIC) and minimum bactericidal concentration (MBC) against *Xhg* using a dose range of 6.25–100 µM (Fig. 4). Compound **X12-k** exhibited an MIC of 25 µM, at which there was no visible bacterial growth observed. However, sub-culturing from the 25 µM MIC well yielded viable colonies on YDC agar, indicating that the compound was not bactericidal at this concentration. In contrast, at 50 µM, no colonies were recovered after plating, confirming bactericidal activity. Thus, **X12-k** exhibits an MIC of 25 µM and an MBC of 50 µM against *Xhg*. In comparison, **X2-c** and **X12-l** showed slightly reduced potency, with MICs of 50 µM and MBCs of 100 µM. These results confirm **X12-k** as the most potent compound, demonstrating two-fold greater inhibitory and bactericidal activity than **X2-c** and **X12-l**.

**Fig. 5:**
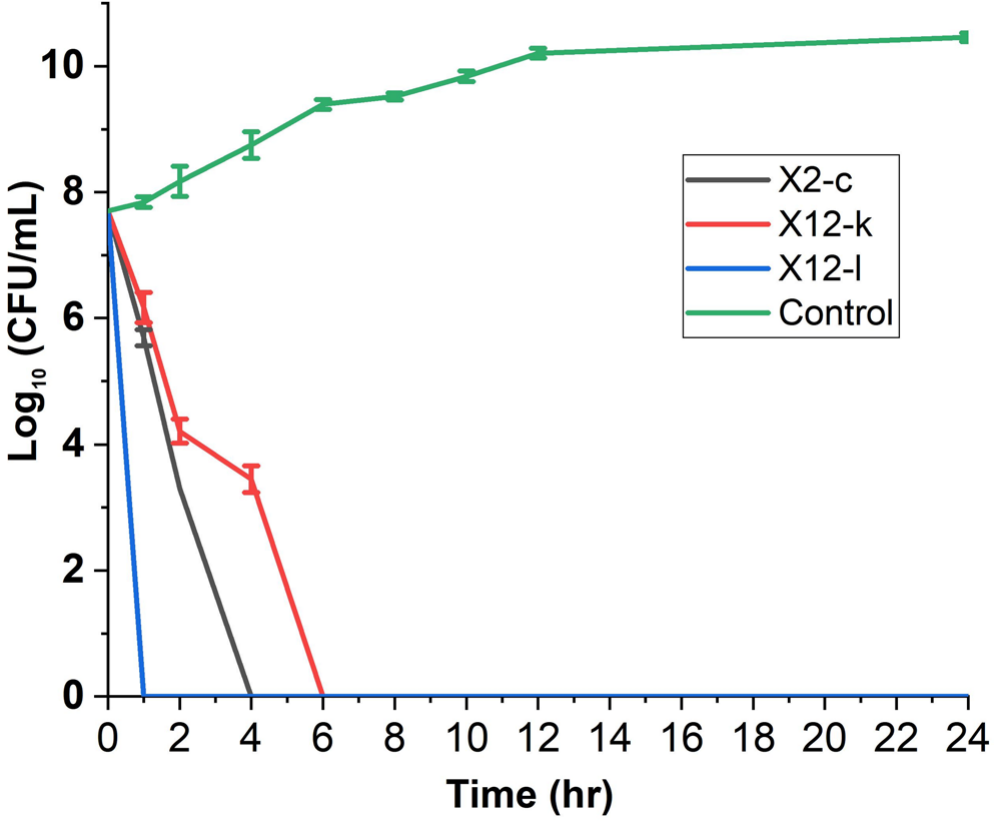
Time–kill kinetics assay evaluating the bactericidal activity of **X2-c**, **X12-k**, and **X12-l** against *Xhg*. *Xhg* cultures were treated with the top 3 compounds (MBCs) **X2-c** (100 µM), **X12-k** (50 µM), and **X12-l** (100 µM) and at the indicated time points, aliquots were collected, serially diluted, and plated on agar to determine viable bacterial counts, which were expressed as log₁₀ (CFU/mL).The untreated culture served as the control. Data are presented as mean ± SD from two independent experiments with two technical replicates in each assay

Furthermore, a time–kill kinetics assay was conducted using a plate-based antibacterial experiment to evaluate the onset time of the bactericidal activity of the top 3 compounds **X2-c**, **X12-k**, and **X12-l** against *Xhg* at their respective MBC concentrations (Fig. 5). As shown in Fig. 5, all three compounds exhibited time-dependent bactericidal activity against *Xhg*. Among them, **X12-l** demonstrated the most rapid antibacterial effect, achieving complete bacterial killing within 1 h of incubation, whereas the untreated control culture showed 7.84 ± 0.08 log CFU/mL at the same time point. In contrast, **X2-c** and **X12-k** showed a gradual reduction in bacterial counts, with complete killing observed after 4 h and 6 h of incubation, respectively. Meanwhile, the untreated control culture continued to proliferate, reaching 8.75 ± 0.21 log CFU/mL at 4 h and 9.40 ± 0.07 log CFU/mL at 6 h, indicating sustained bacterial growth in the absence of treatment. These results indicated that the lead compounds exert potent bactericidal effects against *Xhg* with distinct killing kinetics, with **X12-l** displaying the fastest onset of activity.

**Table 1:** The growth inhibitory effects of synthesized compounds against *Xhg* SM775-12

Small molecules	Core molecular group	R1 or R2	Percentage inhibition						
			25 µM	50 µM		100 µM		200 µM	
X2^*^			< 10	< 10		27 ± 6.4		85 ± 5.0	
X2-a^*^			N/T	N/T		< 10		< 10	
X2-b			< 10	18 ± 2.8		23 ± 3.2		N/T	
X2-c			40 ± 8.4	100 ± 2.1		100 ± 1.1		N/T	
X2-d			10	30 ± 7.4		98 ± 5.2		N/T	
X2-e			N/T	N/T		< 10		N/T	
X5 ^*^			< 10	16 ± 7.5		100 ± 1.1		N/T	
X5-a^*^			< 10	13 ± 4.2		86 ± 3.8		N/T	
X5-b			< 10	< 10		58 ± 2.1		N/T	
X5-c			< 10	< 10		53 ± 5.7		N/T	
X12^*^			< 10	< 10		100 ± 2.3		100 ± 1.8	
X12-a^*^			N/T	N/T		< 10		N/T	
X12-b			N/T	N/T		< 10		N/T	
X12-c			N/T	N/T		< 10		N/T	
X12-d			N/T	N/T		< 10		N/T	
X12-e			N/T	N/T		< 10		N/T	
X12-f			N/T	N/T		19 ± 5.1		N/T	
X12-g			< 10	21 ± 5.7		34 ± 3.9		N/T	
X12-h			< 10	< 10		28 ± 7.6		N/T	
X12-i			< 10	16 ± 2.8		26 ± 4.3		N/T	
X12-j			< 10	< 10		35 ± 4.31		N/T	
X12-k			100 ± 1	100 ± 0.4		100 ± 0.6		N/T	
X12-l			30 ± 2.3	100 ± 1.7		100 ± 0.5		N/T	

^*^Commercially obtained compounds. The antimicrobial inhibition was calculated as the mean of three independent biological replicates

### Structure-activity relationship

The Structure-Activity Relationship (SAR) was studied by altering the core heterocycles of lead compounds **X2**,** X5**, and **X12** to identify active, potent antimicrobial inhibitors, with the aim of understanding how these structural changes influence their antimicrobial activity against *Xhg*. When the compound **X2-a**, an analog of **X2** bearing the phenylpyrimidine moiety, was screened on *Xhg*, it exhibited less than 10% inhibitory activity. Consequently, the core moiety 2-(naphthalen-1-yl)pyrimidine of **X2** was conjugated with imidazole via an isopropyl alcohol linker, yielding derivative **X2-b**. However, neither compound showed anti-*Xanthomonas* activity, with only ≤ 23% inhibition at 100 µM. In contrast, the derivative **X2-c**, which has the conjunction of 4-(2-(piperidin-2-yl)ethyl)phenol linked with 2-(naphthalen-1-yl)pyrimidine, demonstrated enhanced antimicrobial activity, achieving 100% inhibition at 50 µM. Further structural modification, replacing the phenol moiety of **X2-c** with a pyridine moiety (2-(2-(piperidin-2-yl)ethyl)pyridine), resulted in **X2-d**, which retained 98% antimicrobial inhibition but at a higher concentration (100 µM). Interestingly, both **X2-c** and **X2-d** were more active than the parent compound **X2**, highlighting the importance of the conjugated substituents for antimicrobial activity. The conjugated moiety, 4-(2-(piperidin-2-yl)ethyl)phenol, was derived from compound **X5**, which showed 100% antimicrobial inhibition at 100 µM. The enhanced activity may be attributed to the presence of a piperidine ring, especially when paired with a lipophilic alkyl chain and a phenol group. We speculate that piperidine and the alkyl chain may improve membrane permeability, while the phenol moiety facilitates hydrogen bonding interactions, perhaps interfering with bacterial cell membrane disruption or interference with critical microbial processes [40, 41]. In contrast to **X5**, its derivatives (**X5-a** to **X5-c**) bearing the electron-withdrawing groups (e.g., acetoxy or fluorine) on the benzene ring of the 2-phenyloxazole scaffold did not improve inhibitory activity (< 100% inhibition), whereas an electron-donating group (e.g., methoxy) was favorable for enhancing growth inhibition.

Despite the structural modifications of the parent compound **X12** by nitro-imidazole and other phenol derivatives, analogues (**X12-b** to **X12-f**) revealed no significant improvement in activity (≤ 19% inhibition). Even the introduction of a benzimidazole salt pharmacophore into **X12-a** did not affect inhibitory potency. However, replacement of the imidazole core with a carbazole skeleton, followed by conjugation with phenol or other heterocyclic systems including imidazole, morpholine, and triazole (**X12-g** to **X12-j**), demonstrated slightly enhanced activities (≤ 35%) at 100 µM. Interestingly, **X12-k** and **X12-l**, incorporating carbazole conjugated with either 4-(2-(piperidin-2-yl)ethyl)phenol or 2-(2-(piperidin-2-yl)ethyl)pyridine) exhibited significantly enhanced antimicrobial activities (100% inhibition) at 100 µM. Especially, **X12-k** demonstrated complete (100%) *Xhg* growth inhibition at the much lower concentration of 25 µM.

**Fig. 6:**
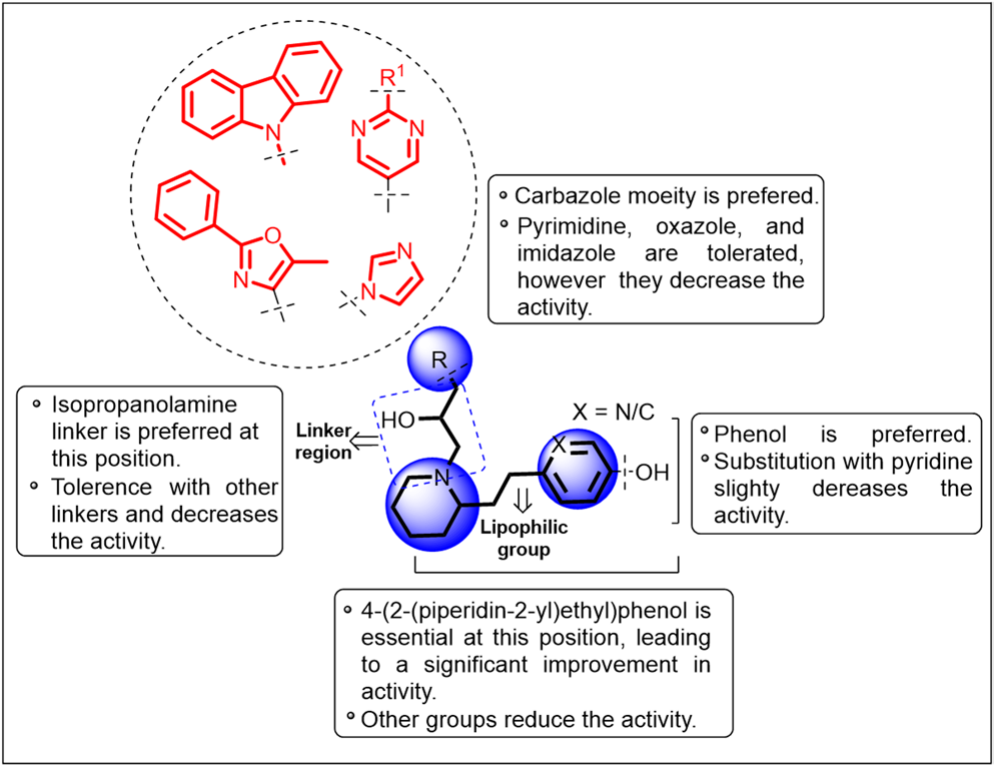
The SAR map of the active compounds. The map was generated on the compounds exhibiting more than 50% inhibitory activity against *Xhg*

As summarized in Table 1, the compounds **X2-c**, **X2-d**, **X5** derivatives, **X12**, **X12-k**, and **X12-l** exhibited more than 50% bacterial growth inhibition across a concentration range of 25–100 µM. Based on these antimicrobial active compounds, an SAR map was designed, revealing that the carbazole moiety is the most favorable heterocyclic core for antibacterial activity, as evidenced by compounds **X12-k** and **X12-l**, which exhibited 100% bacterial inhibition even at 25 and 50 µM respectively (Fig. 6). Though, the compound **X2-c** bearing the pyrimidine moiety also demonstrated 100% bacterial inhibition up to 50 µM, it did not show further improvement in activity below 50 µM. In contrast, the compounds oxazole or imidazole cores were tolerated but demonstrated comparatively reduced antibacterial activity, particularly at concentrations below 100 µM, as observed for other active compounds **X2-c**, **X2-d**, and the **X5** derivatives. Regarding the linker region, the isopropanolamine linker was preferred (**X2-c**, **X2-d**, **X12-k**, and **X12-l)**, likely due to its ability to provide optimal spatial orientation and favorable hydrogen-bonding interactions [41–44]. Although alternative linkers, such as methyl (as in the **X5** derivatives), were tolerated, they resulted in diminished antibacterial efficacy at lower concentrations than 100 µM. Across the compound series, the presence of the 4-(2-(piperidin-2-yl)ethyl)phenol or 2-(2-(piperidin-2-yl)ethyl)pyridine was identified as a critical determinant of activity, as all compounds (**X2-c**, **X2-d**, **X5** derivatives, **X12-k**, and **X12-l**) bearing this substituent exhibited antibacterial effects. Modification at this position with other moieties, such as benzene group, pyrazole, imidazole, morpholine, and triazole reduced the bacterial potency. Notably, the phenolic group (**X2-c**,** X12-k)** was more favorable than pyridine (**X2-d**,** X12-l)**, as substituting phenol for pyridine led to a modest decrease in activity at lower doses, indicating sensitivity to both electronic and steric factors. Thus, these findings demonstrate that the combination of a carbazole core, an isopropanolamine linker, and a 4-(2-(piperidin-2-yl)ethyl)phenol gives the best antibacterial activity, as in compound **X12-k**, which displayed the highest antimicrobial potency, with a MIC of 25 µM and a MBC of 50 µM.

Although the mechanism of the antimicrobial actions of these compounds is unknown, Tian et al. reported that carbazole derivatives bearing an isopropanolamine moiety can enhance membrane permeability in *Xanthomonas oryzae*, resulting in the accumulation of reactive oxygen species (ROS), disruption of the redox balance, inhibition of normal bacterial growth, and ultimately bacterial cell death [41]. Since the lead compound **X12-k** also contains a carbazole scaffold functionalized with an isopropanolamine group, we speculate that it may exhibit a similar membrane-targeting mechanism for *Xhg* inhibition. However, to validate this hypothesis, future studies will focus on comprehensive mechanistic investigations of the most active compounds, including Propidium Iodide (PI) staining and Live/Dead assays to confirm membrane disruption, as well as Scanning Electron Microscopy (SEM) to evaluate morphological alterations in bacterial cells.

**Fig. 7:**
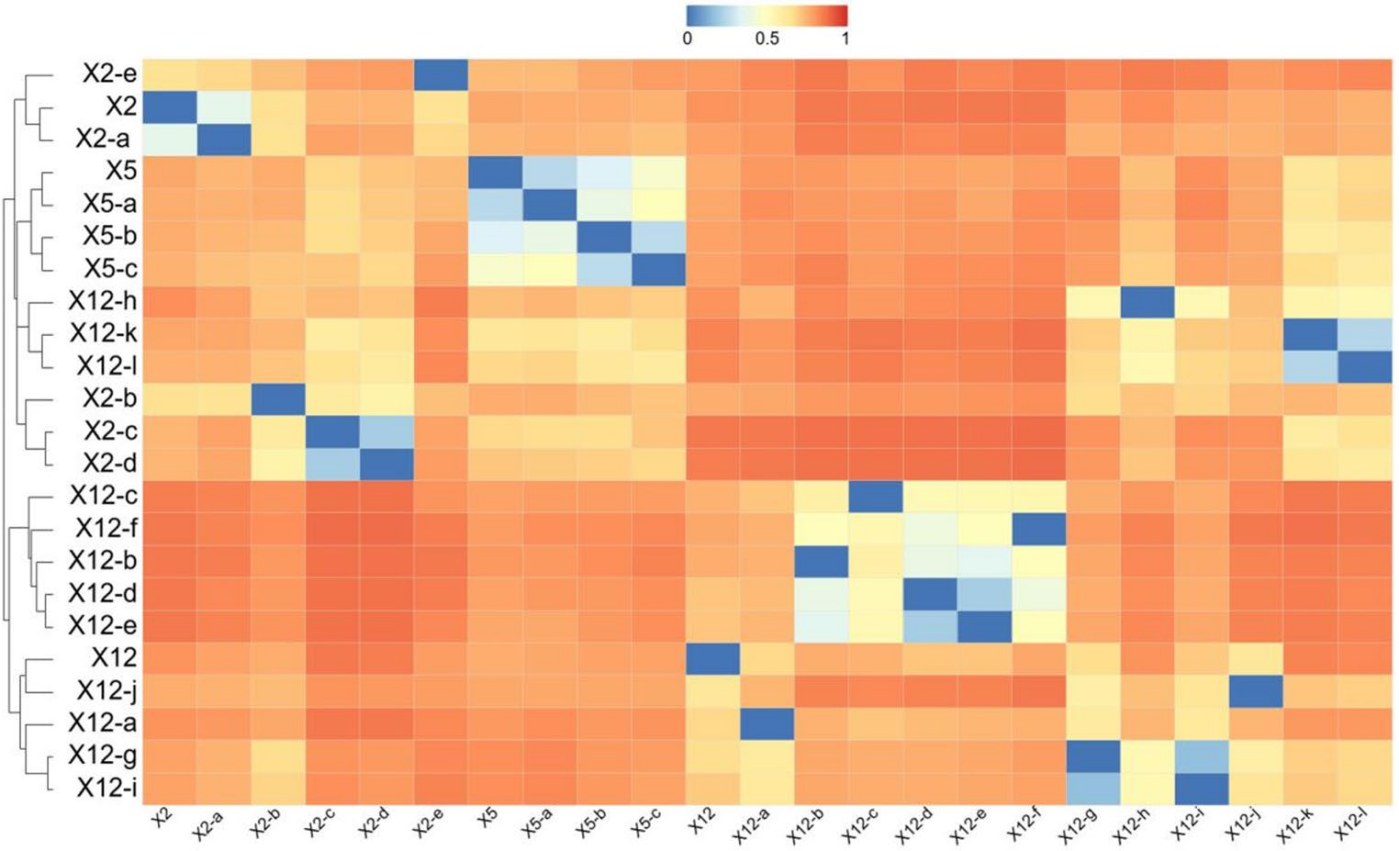
Hierarchical clustering of the **X2**,** X5**, and **X12** analogs based on 2D-Tanimoto similarity scoring. 2D-Tanimoto scoring assesses the structural similarity between two compounds based on the number of shared components. A similarity score ≥ 0.75 indicates a highly similar compound

The structural similarity of the synthesized derivatives was compared with the parent compounds **X2**, **X5**, and **X12** using hierarchical clustering based on 2D Tanimoto similarity scores (calculated via ChemMine, Fig. 7). Most of the derivatives exhibited high similarity to their respective parent scaffolds (Tanimoto coefficient, T ≥ 0.75). However, compounds **X2-c**, **X2-d**, **X12-k**, and **X12-l** showed moderate divergence (T < 0.75) due to the introduction of distinct moieties, specifically, 4-(2-(piperidin-2-yl)ethyl)phenol and 2-(2-(piperidin-2-yl)ethyl)pyridine, which significantly diverged their structural profiles from those of **X2** and **X12**. Furthermore, the presence of a piperidine-linked hydroxypropyl group, which is absent in **X2** and **X12** but incorporated in **X2-c**, **X2-d**, **X12-k**, and **X12-l**, represents a key structural modification that appears to enhance bacterial growth inhibition [40].

### The physicochemical and ADME evaluation of lead compounds

**Table 2:** In silico predicted physicochemical parameters of lead compounds

Compounds	Chemical formula	MW	NRB	HBA	HBD	MR	TPSA	Log *P*	Log S	Lipinski # violation
**X2-c**	C_31_H_35_N_3_O_3_	497.63	10	6	2	151.46	78.71	2.88	− 5.89	0
**X12-k**	C_28_H_32_N_2_O_2_	428.57	7	3	2	136.34	48.63	3.88	− 5.98	0
**X12-l**	C_27_H_31_N_3_O	413.55	7	3	1	132.11	41.29	3.41	− 5.47	0

MW, molecular weight; NRB, no of rotation bonds; HBA, hydrogen bond acceptor; HBD, hydrogen bond donor; MR, molar refractivity; TPSA, topological polar surface area; Log *P*, lipophilicity; Log S, aqueous solubility. The prediction was conducted through SwissADME (https://www.swissadme.ch/)

**Table 3:** In silico prediction of pharmacokinetic profiles for lead compounds

Parameter	X2-c	X12-k	X12-l
*Absorption*			
GI absorption	High	High	High
Skin permeability (log Kp)	− 5.75 cm/s	− 4.97 cm/s	− 5.36 cm/s
Bioavailability score	0.55	0.55	0.55
*Distribution*			
VDss (human) (log L/kg)^*^	0.519	0.74	1.17
BBB permission	No	Yes	Yes
*Metabolism*			
CYP2D6 inhibitor	Yes	Yes	Yes
CYP3A4 inhibitor	Yes	Yes	Yes
Excretion*			
Total clearance	1.022	1.16	1.254

VDss, volume of distribution at steady state; BBB, blood-brain barrier permeability. The prediction was conducted through SwissADME (https://www.swissadme.ch/). *The prediction was conducted through pKCSM-pharmacokinetics (https://biosig.lab.uq.edu.au/pkcsm/)

As part of this medicinal chemistry approach, the drug-likeness of the most potent compounds (**X2-c**, **X12-k**, and **X12-l**) was evaluated through in silico prediction of physicochemical and pharmacokinetic properties using SwissADME and pkCSM (Tables 2 and 3) [48, 49]. All compounds possess molecular weights below 500 Da (413.55–497.63 Da), consistent with favorable oral permeability and their moderate molecular flexibility (7–10 rotatable bonds), along with acceptable hydrogen-bonding characteristics (Hydrogen bonding acceptor = 3–6; Hydrogen bond donor = 1–2), fully comply with Lipinski’s rule of five and support efficient target engagement without excessive polarity [50]. Topological polar surface area (TPSA) values ranging from 41.29 to 78.71 Å² indicate good membrane permeability, with **X12-k** and **X12-l** expected to exhibit enhanced cellular penetration. Moderate lipophilicity (Log *P* = 2.88–3.88) further supports membrane interaction, a desirable feature for antimicrobial agents, although low predicted aqueous solubility (Log *S* ≈ − 5.5 to − 6.0) suggests that future formulation optimization or synthesis of salts may be required [51]. Furthermore, the pharmacokinetic predictions indicate uniform bioavailability of 0.55 across all compounds, and a low rate of skin permeability (log Kp ≈ − 5.0 to − 5.8 cm/s). The distribution analysis revealed moderate to high volumes of distribution (VDss, log L/kg = 0.51–1.17), with **X12-l** showing greater tissue distribution. Additionally, it was demonstrated that compounds **X12-k** and **X12-l** can permeate the blood-brain barrier (BBB), whereas compound **X2-c** is unlikely to cross the BBB, potentially reducing the risk of central nervous system–related side effects. All compounds were predicted to inhibit CYP2D6 and CYP3A4, suggesting the need for further experimental validation. Moreover, the predicted total clearance rates for all compounds ranged from 1.02 to 1.25, indicating moderate clearance. Overall, these in silico analyses demonstrate that the lead compounds possess a favorable balance of molecular size, polarity, lipophilicity, and oral absorption, supporting their progression as drug-like antimicrobial candidates [52]. While solubility limitations, CYP inhibition, and BBB permeability (for **X12-k** and **X12-l**) represent potential challenges, the absence of Lipinski violations and acceptable ADME profiles justify further biological evaluation and rational lead optimization [53].

## Conclusion

In this study, we synthesized and evaluated a series of **X2**, **X5**, and **X12** derivatives for their growth inhibitory activity against *Xhg* growth. Three of them, **X2-c**, **X12-k**, and **X12-l**, exhibited significantly enhanced antibacterial activity, with lower MIC and MBC than their parent compounds. We identified certain scaffolds, such as carbazole and 4-(2-(piperidin-2-yl)ethyl)phenol, from initial hits, which, when combined into a single molecular framework, may help enhance antibacterial activity. Notably, **X12-k** demonstrated significant antimicrobial effect, with an MIC of 25 µM (potency) and an MBC of 50 µM (efficacy), and is a promising lead compound for the management of bacterial tomato leaf spot. **X12-l** at its MBC (100 µM) showed the rapid bactericidal effect against *Xhg*, achieving complete killing within 1 h, thereby providing strong evidence of antibacterial bioactivity. Furthermore, favorable in silico drug-likeness and ADME profiles of the lead compounds **X2-c**, **X12-k**, and **X12-l** support their continued biological evaluation and rational optimization, highlighting their potential as promising alternatives to conventional antibiotics. In future studies, the antibacterial spectrum of the lead compounds will be evaluated against multiple isolates of *Xhg* and *X. perforans*. Additional investigations will include honeybee toxicity assessments, in vitro antibiofilm activity against biofilm-embedded *Xhg*, and evaluation of antimicrobial resistance development. Mechanistic insights into bacterial cell integrity disruption will be obtained through microscopic analyses. Antimicrobial efficacy will also be assessed on tomato seeds artificially or naturally infested with *Xhg*. Furthermore, *in planta* efficacy will be examined in the tomato seedling model using both preventive and curative treatment approaches. Three-week-old tomato seedlings will be inoculated by foliar spraying with *Xhg*, followed by treatment with the lead molecules 1 day prior to infection for preventive evaluation or at 2 days post-infection (dpi) for curative assessment, as previously reported [32]. Disease severity will be assessed at 7 dpi, and bacterial populations will be quantified. Additionally, evaluation of their activity against other phytopathogenic bacteria and the development of successful formulation approaches to advance these compounds for field application, with the goal of improving food security, will be undertaken.

## Materials and methods

### Chemistry section

All reagents and starting materials were procured commercially from Sigma-Aldrich Inc., Fisher Scientific, or Combi-blocks. The compounds 1-(5-methyl-1-phenyl-1*H*-pyrazol-4-yl)-*N*-((2-(naphthalen-1-yl)pyrimidin-5-yl)methyl)ethan-1-amine (**X2**), 1-(5-methyl-1-phenyl-1*H*-pyrazol-4-yl)-*N*-((2-(o-tolyl)pyrimidin-5-yl)methyl)methanamine (**X2-a**), 4-(2-(1-((2-(4-methoxyphenyl)-5-methyloxazol-4-yl)methyl)piperidin-2-yl)ethyl)phenol (**X5**), methyl 4-(5-((2-(4-hydroxyphenethyl)piperidin-1-yl)methyl)-4-methyloxazol-2-yl)benzoate (**X5-a**), 1-(1*H*-imidazol-1-yl)-3-phenoxypropan-2-yl 4-methoxybenzoate (**X12**), and 3-(2-hydroxy-3-phenoxypropyl)-1-methyl-1*H*-benzo[d]imidazol-3-ium iodide (**X12-a**) were also obtained from Chembridge. The commercially available starting material, epichlorohydrin, was used as a racemic mixture of enantiomers. Consequently, the synthesized products were expected to be obtained as enantiomeric mixtures, and therefore, chiral HPLC or optical rotation analyses were not performed in this study. However, our future studies will explore optical activity. Merck precoated silica gel plates (Art. 5554) treated with a fluorescent indicator were used for analytical thin-layer chromatography (TLC). Purification of intermediates and final compounds was performed by silica gel column chromatography using silica gel 9385 (Merck) and methanol, ethyl acetate, and hexane were used as the solvents. ^1^H NMR spectra were recorded on a VNMR (400 MHz) spectrometer at the Department of Pharmacy, University of Findlay. The NMR spectra were recorded in CDCl_3_ using = $\delta$ 7.24 (1H) ppm, DMSO-*d*_*6*_ using $\delta$ = 2.50 (1H) ppm, and Acetone-d_*6*_ using $\delta$ = 2.05–2.22 (^1^H) ppm as reference for the residual solvent chemical shifts. All chemical shifts ($\delta$) are expressed in units of ppm and *J* values are given in Hz. Multiplicities are abbreviated as follows: s = singlet, d = doublet, t = triplet, m = multiplet or overlap of nonequivalent resonances, and dd = doublet of doublets. Mass spectrometry (MS) was performed using a LCQ DECA-XP (Thermo Fisher) at the College of Pharmacy, University of Findlay.

*Synthesis of (2-(naphthalen-1-yl)pyrimidin-5-yl)methanol* (**2**). Synthesis parameters were adjusted from Chaikin et al. [46]. 2-(naphthalen-1-yl)pyrimidine-5-carbaldehyde (2 g, 8.4 mmol, 1 equiv.) was dissolved in dry methanol (30 mL) and cooled to 0 °C. NaBH_4_ (1.6 g, 2 equiv.) was dissolved in methanol and added dropwise to the solution. The reaction mixture was stirred for 2 h and the reaction was quenched by adding water after confirmation with TLC. The product was extracted with ethyl acetate, and the combined organic layers were dried over anhydrous sodium sulfate. After solvent evaporation, the final product was obtained as an off-white powder in 82% yield. The product was confirmed by mass spectrometry (MS).

*Synthesis of 1-chloro-3-((2-(naphthalen-1-yl)pyrimidin-5-yl)methoxy)propan-2-ol* (**3**). Synthesis parameters were adjusted from the published literature [47]. (2-(naphthalen-1-yl)pyrimidin-5-yl)methanol (1 g, 4.2 mmol, 1 equiv.) and KOH (235 mg, 1 equiv.) were dissolved in acetonitrile (20 mL). Epichlorohydrin (579 mg, 1.5 equiv.) was added dropwise to the solution. The reaction mixture was stirred for 6 h at 60 °C. The progress of the reaction was followed by TLC analysis. After completion, the reaction mixture was cooled to room temperature. After completion of the reaction, the volatiles were removed in vacuo, and the residue was purified by silica gel column chromatography using 4:6 (Hex: EtOAc) to obtain the desired product **3** in 70% yield as a viscous liquid. The product was confirmed by mass spectrometry (MS).

*Synthesis of 1-(1H-imidazol-1-yl)-3-((2-(naphthalen-1-yl)pyrimidin-5-yl)methoxy)propan-2-ol* (**X2-b**). The starting material **3** (164 mg, 0.5 mmol), imidazole (51 mg, 1.5 equiv.), and K_2_CO_3_ (69 mg, 1 equiv.) were dissolved in acetonitrile (5 mL). The reaction mixture was refluxed for 6 h, and the reaction progress was monitored by TLC. Upon completion, the reaction mixture was cooled to room temperature, the solvents were removed in vacuo, and the residue was purified by silica gel column chromatography using 1:9 (Hex: EtOAc) to obtain the desired product **X2-b** as a solid in 65% yield. ^1^H NMR (400 MHz, CDCl_3_): *δ* 8.9 (2 H, s), 8.57 (1H, t, *J* = 5.2 Hz), 8.05–7.97 (4 H, m), 7.60 (1H, t, *J* = 8 Hz), 7.55–7.51 (4 H, m), 5.2 (2 H, m), 4.6 (2 H, s), 4.09 (1H, d, *J* = 10.8 Hz), 3.94–3.89 (3 H, m). LC-MS (ESI): m/z 361 [M + H]⁺ (calculated for C_21_H_20_N_4_O_2_: 360).

*Synthesis of 4-(2-(1-(2-hydroxy-3-((2-(naphthalen-1-yl)pyrimidin-5- yl)methoxy) propyl)piperidin-2-yl)ethyl)phenol* (**X2-c**). The starting material **3** (164 mg, 0.5 mmol), 4-(2-(piperidin-2-yl)ethyl)phenol (153 mg, 1.5 equiv.), and K_2_CO_3_ (69 mg, 1 equiv.) were dissolved in acetonitrile (5 mL). The reaction mixture was refluxed for 8 h, and the reaction progress was monitored by TLC. Upon completion, the reaction mixture was cooled to room temperature, the solvents were removed in vacuo, and the residue was purified by silica gel column chromatography in 2:8 (MeOH: EtOAc) to obtain the desired product **X2-c** as a sticky solid in 60% yield. ^1^H NMR (400 MHz, DMSO-*d*_*6*_): *δ* 9.04 (1H, s), 8.94 (2 H, d, *J* = 5.2 Hz), 8.56 (1H, d, *J* = 7.6 Hz), 8.04–7.97 (3 H, m), 7.62–7.50 (3 H, m), 6.92–6.89 (2 H, m), 6.59–6.56 (2 H, m), 4.62 (2 H, d, *J* = 4.8 Hz), 3.53–3.38 (4 H, m), 2.62 (3 H, m), 2.35–2.28 (4 H, m), 1.68–1.26 (8 H, m). LC-MS (ESI): m/z 498 [M + H]⁺ (calculated for C_31_H_35_N_3_O_3_: 497).

*Synthesis of 1-((2-(naphthalen-1-yl)pyrimidin-5-yl)methoxy)-3-(2-(2-(pyridin-2- yl)ethyl)piperidin-1-yl)propan-2-ol* (***X2-d***). The starting material **3** (164 mg, 0.5 mmol), 2-(2-(piperidin-2-yl)ethyl)pyridine (142 mg, 1.5 equiv.), and K_2_CO_3_ (69 mg, 1 equiv.) were dissolved in acetonitrile (5 mL). The reaction mixture was refluxed for 8 h, and the reaction progress was monitored by TLC. Upon completion, the reaction mixture was cooled to room temperature, the solvents were removed in vacuo, and the residue was purified by silica gel column chromatography using 2:8 (MeOH: EtOAc) to obtain the desired product **X2-d** as a sticky solid in 55% yield. ^1^H NMR (400 MHz, DMSO-d_*6*_): *δ* 8.93 (2 H, s), 8.55 (1H, d, *J* = 6.8 Hz), 8.38 (1H, m), 8.3 (2 H, d, *J* = 8.4 Hz), 7.97 (1H, d, *J* = 7.2 Hz), 7.62–7.56 (2 H, m), 7.54–7.51 (2 H, m), 7.19–7.15 (1H, m), 7.09–7.08 (1H, m), 4.65–4.57 (2 H, m), 3.73 (1H, m), 3.55–3.54 (2 H, m), 3.52–3.48 (2 H, m), 2.76–2.69 (2 H, m), 2.67–2.60 (3 H, m), 1.85–1.56 (5 H, m), 1.4–1.17 (4 H, m). LC-MS (ESI): m/z 483.4 [M + H⁺] (calculated for C_30_H_34_N_4_O_2_: 482.27).

*Synthesis of (2-(naphthalen-1-yl)pyrimidin-5-yl)methyl 3*,*4*,*5-trimethoxybenzoate* (**X2-e**). The starting material **2** (117 mg, 0.5 mmol), 3,4,5-trimethoxybenzoyl chloride (172 mg, 1.5 equiv.), and KOH (28 mg, 1 equiv.) were dissolved in ethanol (5 mL). The reaction mixture was refluxed for 5 h, and the reaction progress was monitored by TLC. Upon completion, the reaction mixture was cooled to room temperature, the solvents were removed in vacuo, and the residue was purified by silica gel column chromatography using 3:7 (Hex: EtOAc) to obtain the desired product **X2-e** as a white solid in 55% yield. ^1^H NMR (400 MHz, Acetone-*d*_*6*_): *δ* 9.1 (1H, s), 8.85–8.82 (1H, m), 8.19 (1H, dd, *J* = 7.2, 1.8 Hz), 8.06 (1H, d, *J* = 8.0 Hz), 8.01–7.99 (1H, m), 7.64 (1H, t, *J* = 8.0 Hz), 7.57–7.53 (2 H, m), 7.46–7.45 (1H, m), 7.4 (2 H, s), 5.56 (2 H, s), 3.94–3.83 (6 H, m), 3.80–3.71 (3 H, m). LC-MS (ESI): m/z 833 [2 M + Na⁺] (calculated for C_25_H_22_N_2_O_5_: 430).

*Synthesis of 4-(2-(1-((2-(2-fluorophenyl)-5-methyloxazol-4-yl)methyl)piperidin-2- yl)ethyl)phenol* (**X5-b**). 4-(chloromethyl)-2-(2-fluorophenyl)-5-methyloxazole **4** (112 mg, 0.5 mmol), 4-(2-(piperidin-2-yl)ethyl)phenol (153 mg, 1.5 equiv.), and K_2_CO_3_ (69 mg, 1 equiv.) were dissolved in acetonitrile (5 mL). The reaction mixture was refluxed for 7 h, and the reaction progress was monitored by TLC. Upon completion, the reaction mixture was cooled to room temperature, the solvents were removed in vacuo, and the residue was purified by silica gel column chromatography using 2:8 (MeOH: EtOAc) to obtain the desired product **X5-b** as a sticky solid in 56% yield. ^1^H NMR (400 MHz, CDCl_3_): *δ* 8.0 (1H, t, *J* = 3.2 Hz), 7.41–7.34 (1H, m), 7.26–7.13 (3 H, m), 7.04 (1H, d, *J* = 8.0 Hz), 6.92 (1H, d, *J* = 8.4 Hz), 6.72 (1H, d, *J* = 8.0 Hz), 4.9 (1H, m), 3.80 (1H, d, *J* = 14.4 Hz), 3.54–3.51 (1H, m), 2.89 (1H, d, *J* = 11.2 Hz), 2.73–2.48 (3 H, m), 2.44–2.39 (4 H, m), 2.29 (1H, m), 2.10–2.04 (1H, m) 1.90–1.76 (1H, m), 1.68 (2 H, d, *J* = 16.8 Hz), 1.57 (3 H, m). LC-MS (ESI): m/z 395 [M + H]⁺ (calculated for C_24_H_27_FN_2_O_2_: 394).

*Synthesis of 2-(2-fluorophenyl)-5-methyl-4-((2-(2-(pyridin-2-yl)ethyl)piperidin-1-yl) methyl)oxazole* (**X5-c**). 4-(chloromethyl)-2-(2-fluorophenyl)-5-methyloxazole **4** (112 mg, 0.5 mmol), 2-(2-(piperidin-2-yl)ethyl)pyridine (142 mg, 1.5 equiv.), and K_2_CO_3_ (69 mg, 1 equiv.) were dissolved in acetonitrile (5 mL). The reaction mixture was refluxed for 7 h, and the reaction progress was monitored by TLC. Upon completion, the reaction mixture was cooled to room temperature, the solvents were removed in vacuo, and the residue was purified by silica gel column chromatography using 2:8 (MeOH: EtOAc) to obtain the desired product **X5-c** as a sticky solid in 45% yield. ^1^H NMR (400 MHz, DMSO-d_*6*_): *δ* 8.40 (1H, d, *J* = 4.8 Hz ), 7.89 (1H, t, *J* = 8.0 Hz), 7.60 (1H, t, *J* = 7.6 Hz), 7.51–7.46 (1H, m), 7.34–7.28 (1H, m), 7.24 (2 H, t, *J* = 7.2 Hz), 7.11 (1H, t, *J* = 7.2 Hz), 3.7 (1H, m), 2.82–2.76 (2 H, m), 2.66–2.63 (2 H, m), 2.44–2.35 (3 H, m), 2.18–1.85 (2 H, m), 1.60–1.57 (2 H, m), 1.47–1.41 (4 H, m), 1.21–1.16 (2 H, m). LC-MS (ESI): m/z 380.3 [M + H]⁺ (calculated for C_23_H_26_FN_3_O: 379.21).

*Synthesis of 1-chloro-3-(5-nitro-1H-imidazol-1-yl)propan-2-ol* (**7**). Synthesis parameters were adjusted from Katherine et al. [40]. 5-nitro-1*H*-imidazole (**6**) (1 g, 8.8 mmol), and KOH (492 mg, 1 equiv.) were dissolved in DMF (20 mL). Epichlorohydrin (1.2 g, 1.5 equiv.) was added dropwise to the solution. The reaction mixture was stirred for 6 h at 60 °C. The progress and completion of the reaction were monitored through TLC. After completion and cooling to room temperature, the product was extracted three times with ethyl acetate (3 × 20 mL). The combined extracts were dried over anhydrous magnesium sulfate, filtered, and concentrated to yield product **7** as a solid in 85% yield. The product was confirmed by mass spectrometry (MS).

* Synthesis of 1-(9H-carbazol-9-yl)-3-chloropropan-2-ol* (**9**). Synthesis parameters were adjusted from Katherine et al. [43]. Carbazole (**8**) (1 g, 5.9 mmol), and KOH (492 mg, 1 equiv.) were dissolved in DMF (20 mL). Epichlorohydrin (1.2 g, 1.5 equiv.) was added dropwise to the solution. The reaction mixture was stirred for 6 h at 60 °C. The progress and completion of the reaction were monitored through TLC. After completion and cooling to room temperature, the product was extracted three times with ethyl acetate (3 × 20 mL). The combined extracts were dried over anhydrous magnesium sulfate, filtered, and concentrated to yield product **9** as a solid in 85% yield. The product was confirmed by mass spectrometry (MS).

*Synthesis of N-(4-(2-hydroxy-3-(2-methyl-5-nitro-1H-imidazol-1-yl)propoxy) phenyl)acetamide* (**X12-b**). The starting material **7** (102 mg, 0.5 mmol), *N*-(4-hydroxyphenyl)acetamide (113 mg, 1.5 equiv.), and K_2_CO_3_ (69 mg, 1 equiv.) were dissolved in acetonitrile (5 mL). The reaction mixture was refluxed for 7 h, and the reaction progress was monitored by TLC. Upon completion, the reaction mixture was cooled to room temperature, the solvents were removed in vacuo, and the residue was purified by silica gel column chromatography using 1:9 (Hex: EtOAc) to obtain the desired product **X12-b** as a white solid in 78% yield. ^1^H NMR (400 MHz, DMSO-*d*_*6*_): *δ* 9.7 (1H, s), 8.04 (1H, m), 7.4 (2 H, d, *J* = 8.8 Hz), 6.8 (2 H, d, *J* = 8.8 Hz), 5.5 (1H, s), 4.60–4.56 (1H, m), 4.30–4.24 (1H, m), 4.08 (1H, m), 3.9 (2 H, d, *J* = 5.5 Hz), 2.46 (3 H, s), 3.30 (3 H, s). LC-MS (ESI): m/z 335.3 [M + H]⁺ (calculated for C_15_H_18_N_4_O_5_: 334.

*Synthesis of 3-(2-hydroxy-3-(2-methyl-5-nitro-1H-imidazol-1-yl)propoxy)-4-methoxybenzaldehyde* (**X12-c**). The starting material **7** (102 mg, 0.5 mmol), 3-hydroxy-4-methoxybenzaldehyde (114 mg, 1.5 equiv.), and K_2_CO_3_ (69 mg, 1 equiv.) were dissolved in acetonitrile (5 mL). The reaction mixture was refluxed for 7 h, and the reaction progress was monitored by TLC. Upon completion, the reaction mixture was cooled to room temperature, the solvents were removed in vacuo, and the residue was purified by silica gel column chromatography using 1:9 (Hex: EtOAc) to obtain the desired product **X12-c** as a white solid in 70% yield. ^1^H NMR (400 MHz, CDCl_3_): *δ* 9.8 (1H, s), 7.9 (1H, s), 7.5 (1H, d, *J* = 8.4 Hz), 7.45 (1H, s), 7.0 (1H, d, *J* = 8.4 Hz), 4.69–4.66 (1H, m), 4.39–4.37 (2 H, m), 4.26–4.23 (1H, m), 4.13–4.09 (1H, m), 3.9 (3 H, s), 2.9 (1H, m), 2.57 (3 H, s). LC-MS (ESI): m/z 336 [M + H]⁺ (calculated for C_15_H_17_N_3_O_6_: 335).

*Synthesis of 1-(4-(2-hydroxy-3-(2-methyl-5-nitro-1H-imidazol-1-yl)propoxy)phenyl)ethan-1-one* (**X12-d**). The starting material **7** (102 mg, 0.5 mmol), 1-(4-hydroxyphenyl)ethan-1-one (102 mg, 1.5 equiv.), and K_2_CO_3_ (69 mg, 1 equiv.) were dissolved in acetonitrile (5 mL). The reaction mixture was refluxed for 7 h, and the reaction progress was monitored by TLC. Upon completion, the reaction mixture was cooled to room temperature, the solvents were removed in vacuo, and the residue was purified by silica gel column chromatography using 1:9 (Hex: EtOAc) to obtain the desired product **X12-d** as a white solid in 70% yield. ^1^H NMR (400 MHz, DMSO-*d*_*6*_): *δ* 8.0 (1H, s), 7.8 (2 H, d, *J* = 8.8 Hz), 7.0 (2 H, d, *J* = 8.8 Hz), 5.54 (1H, s), 4.56–4.52 (1H, m), 4.25–4.20 (1H, m), 4.08–4.06 (3 H, m), 3.7 (3 H, s), 2.4 (3 H, s). LC-MS (ESI): m/z 320 [M + H]⁺ (calculated for C_15_H_17_N_3_O_5_: 319).

*Synthesis of methyl 4-(2-hydroxy-3-(2-methyl-5-nitro-1H-imidazol-1-yl)propoxy)benzoate* (**X12-e**). The starting material **7** (102 mg, 0.5 mmol), methyl 4-hydroxybenzoate (114 mg, 1.5 equiv.), and K_2_CO_3_(69 mg, 1 equiv.) were dissolved in acetonitrile (5 mL). The reaction mixture was refluxed for 7 h, and the reaction progress was monitored by TLC. Upon completion, the reaction mixture was cooled to room temperature, the solvents were removed in vacuo, and the residue was purified by silica gel column chromatography in 2:8 (MeOH: EtOAc) to obtain the desired product **X12-e** as a white solid in 61% yield. ^1^H NMR (400 MHz, CDCl_3_): *δ* 8.02 (2 H, d, *J* = 9.2 Hz), 7.98 (1H, s), 6.95 (2 H, d, *J* = 8.8 Hz), 4.71–4.68 (3 H, m), 4.39–4.35 (2 H, m), 4.23–4.20 (1H, m), 3.94 (3 H, s), 2.56 (3 H, s). LC-MS (ESI): m/z 336 [M + H]⁺ (calculated for C_15_H_17_N_3_O_6_: 335).

*Synthesis of 1-(4-isopropyl-2-methylphenoxy)-3-(2-methyl-5-nitro-1H-imidazol-1-yl)propan-2-ol* (**X12-f**). The starting material **7** (102 mg, 0.5 mmol), 4-isopropyl-2-methylphenol (112 mg, 1.5 equiv.), and K_2_CO_3_(69 mg, 1 equiv.) were dissolved in acetonitrile (5 mL). The reaction mixture was refluxed for 7 h, and the reaction progress was monitored by TLC. Upon completion, the reaction mixture was cooled to room temperature, the solvents were removed in vacuo, and the residue was purified by silica gel column chromatography using 2:8 (MeOH: EtOAc) to obtain the desired product **X12-f** as a white solid in 55% yield. ^1^H NMR (400 MHz, CDCl_3_): *δ* 7.9 (1H, s), 7.08 (1H, d, *J* = 7.2 Hz), 6.80 (1H, d, *J* = 7.6 Hz), 6.69 (1H, s), 5.39 (1H, s), 4.70 (1H, d, *J* = 12.4 Hz), 4.46–4.40 (2 H, m), 4.20–4.16 (1H, m), 4.08–4.05 (1H, m), 2.81–2.85 (1H, m), 2.57 (3 H, s), 2.2 (3 H, s), 1.30–1.18 (6 H, s). LC-MS (ESI): m/z 334.0 [M + H]⁺ (calculated for C_17_H_23_N_3_O_4_: 333).

*Synthesis of 1-(9H-carbazol-9-yl)-3-(1H-imidazol-1-yl)propan-2-ol* (**X12-g**). The starting material **9** (130 mg, 0.5 mmol), imidazole (51 mg, 1.5 equiv.), and K_2_CO_3_ (69 mg, 1 equiv.) were dissolved in acetonitrile (5 mL). The reaction mixture was refluxed for 8 h, and the reaction progress was monitored by TLC. Upon completion, the reaction mixture was cooled to room temperature, the solvents were removed in vacuo, and the residue was purified by silica gel column chromatography using 2:8 (MeOH: EtOAc) to obtain the desired product **X12-g** as a white solid in 63% yield. ^1^H NMR (400 MHz, DMSO-d_*6*_): *δ* 8.01 (2 H, d, *J* = 7.6 Hz), 7.42–7.37 (4 H, m), 7.22–7.18 (3 H, m), 6.96 (1H, s), 6.73 (1H, m), 4.37–4.26 (4 H, m), 3.89–3.87 (2 H, m). LC-MS (ESI): m/z 292 [M + H]⁺ (calculated for C_18_H_17_N_3_O: 291).

*Synthesis of 1-(9H-carbazol-9-yl)-3-morpholinopropan-2-ol* (**X12-h**). The starting material **9** (130 mg, 0.5 mmol), morpholine (65 mg, 1.5 equiv.), and K_2_CO_2_ (69 mg, 1 equiv.) were dissolved in acetonitrile (5 mL). The reaction mixture was refluxed for 7 h, and the reaction progress was monitored by TLC. Upon completion, the reaction mixture was cooled to room temperature, the solvents were removed in vacuo, and the residue was purified by silica gel column chromatography using 1:9 (MeOH: EtOAc) to obtain the desired product **X12-h** as a white solid in 52% yield. ^1^H NMR (400 MHz, DMSO-d_*6*_): *δ* 8.08 (2 H, d, *J* = 4.0 Hz), 7.57 (1H, d, *J* = 8.4 Hz), 4.27 (1H, dd, *J* = 14.4, 6.8 Hz), 4.04 (1H, m), 3.51 (4 H, m), 2.33–2.31 (6 H, m). MS (ESI): m/z 311 [M + H]⁺ (calculated for C_19_H_22_N_2_O_2_: 310).

*Synthesis of 1-(9H-carbazol-9-yl)-3-(1H-1,2,3-triazol-1-yl)propan-2-ol* (**X12-i**). The starting material **9** (130 mg, 0.5 mmol), 1*H*-1,2,3-triazole (51 mg, 1.5 equiv.), and K_2_CO_3_ (69 mg, 1 equiv.) were dissolved in acetonitrile (5 mL). The reaction mixture was refluxed for 6 h, and the reaction progress was monitored by TLC. Upon completion, the reaction mixture was cooled to room temperature, the solvents were removed in vacuo, and the residue was purified by silica gel column chromatography using 3:7 (MeOH: EtOAc) to obtain the desired product **X12-i** as a white solid in 66% yield. ^1^H NMR (400 MHz, CDCl_3_): *δ* 8.02 (2 H, d, *J* = 8.0 Hz), 7.80 (2 H, d, *J* = 15.6 Hz), 7.44–7.40 (2 H, m), 7.34 (2 H, d, *J* = 8.4 Hz), 7.22–7.16 (2 H, m), 4.52–4.43 (2 H, m), 4.38–4.37 (2 H, m), 4.24–4.12 (1H, m), 4.14–4.08 (1H, m). LC-MS (ESI): m/z 293.1 [M + H]⁺ (calculated for C_17_H_16_N_4_O: 292).

*Synthesis of 1-(9H-carbazol-9-yl)-3-(1H-imidazol-1-yl)propan-2-yl phenylmethanesulfonate* (**X12-j**). The compound **X12-g** (100 mg, 0.34 mmol), phenylmethanesulfonyl fluoride (88 mg, 1.5 equiv.), and K_2_CO_3_ (47 mg, 1 equiv.) were dissolved in acetonitrile (5 mL). The reaction mixture was refluxed for 7 h, and the reaction progress was monitored by TLC. Upon completion, the reaction mixture was cooled to room temperature, the solvents were removed in vacuo, and the residue was purified by silica gel column chromatography using 1:9 (MeOH: EtOAc) to obtain the desired product **X12-j** as a white solid in 63% yield. ^1^H NMR (400 MHz, CDCl_3_): *δ* 8.06 (2 H, d, *J* = 8.0 Hz), 7.48–7.44 (3 H, m), 7.30–7.21 (7 H, m), 7.09 (2 H, m), 7.03 (1H, t, *J* = 6.8 Hz), 6.87–6.88 (1H, m), 5.19–5.16 (1H, m), 4.51–4.46 (1H, m), 4.42–4.36 (1H, m), 4.18 (2 H, d, *J* = 5.2 Hz), 3.64–3.55 (2 H, m). LC-MS (ESI): m/z 445 [M + H]⁺ (calculated for C_25_H_23_N_3_O_3_S: 446).

*Synthesis of 4-(2-(1-(3-(9H-carbazol-9-yl)-2-hydroxypropyl)piperidin-2-yl)ethyl)phenol *(**X12-k**). The starting material **9** (130 mg, 0.5 mmol), 4-(2-(piperidin-2-yl)ethyl)phenol (153 mg, 1.5 equiv.), and K_2_CO_3_ (69 mg, 1 equiv.) were dissolved in acetonitrile (5 mL). The reaction mixture was refluxed for 8 h, and the reaction progress was monitored by TLC. Upon completion, the reaction mixture was cooled to room temperature, the solvents were removed in vacuo, and the residue was purified by silica gel column chromatography in 3:7 (MeOH: EtOAc) to obtain the desired product **X12-k** as a white solid in 49% yield. ^1^H NMR (400 MHz, CDCl_3_): *δ* 8.08–8.01 (2 H, m), 7.48–7.40 (4 H, m), 7.22–7.17 (3 H, m), 6.72–6.50 (3 H, m), 4.46–4.38 (4 H, m), 3.9 (1H, m), 2.9–2.23 (7 H, m), 1.72–1.19 (7 H, m). LC-MS (ESI): m/z 429 [M + H]⁺ (calculated for C_28_H_32_N_2_O_2_: 428).

*Synthesis of 1-(9H-carbazol-9-yl)-3-(2-(2-(pyridin-2-yl)ethyl)piperidin-1-yl)propan-2-ol* (**X12-l**). The starting material **9** (130 mg, 0.5 mmol), 2-(2-(piperidin-2-yl)ethyl)pyridine (142 mg, 1.5 equiv.), and K_2_CO_3_ (69 mg, 1 equiv.) were dissolved in acetonitrile (5 mL). The reaction mixture was refluxed for 7 h, and the reaction progress was monitored by TLC. Upon completion, the reaction mixture was cooled to room temperature, the solvents were removed in vacuo, and the residue was purified by silica gel column chromatography using 3:7 (MeOH: EtOAc) to obtain the desired product **X12-l** as a sticky solid in 48% yield. ^1^H NMR (400 MHz, CDCl_3_): *δ* 8.4 (1H, m), 8.06–8.04 (2 H, m), 7.49–7.43 (4 H, m), 7.22–7.18 (2 H, m), 7.04–6.96 (2 H, m), 6.81 (1H, d, *J* = 7.6 Hz), 4.34–4.20 (4 H, m), 2.90–2.87 (1H, m), 2.74–2.69 (2 H, m), 2.62–2.57 (2 H, m), 2.45–2.34 (2 H, m), 2.10–1.84 (3 H, m), 1.66–1.33 (5 H, m). MS (ESI): m/z 414 [M + H]⁺ (calculated for C_27_H_31_N_3_O: 413).

### Antimicrobial growth inhibition study on *Xanthomonas hortorum* pv. *gardneri*

*Xanthomonas hortorum* pv. *gardneri* (*Xhg*) SM775-12 strain was used as a model strain to identify growth inhibitors. *Xanthomonas* spp. was grown in yeast dextrose calcium carbonate (YDC; Himedia #M1182) agar plate for 28 h at 28 °C. A fresh *Xhg* SM775-12 bacterial culture was grown in diluted Luria-Bertani (LB) broth (25% LB; ThermoFisher Scientific, Waltham, MA), for 28 h at 28 °C with 180 rpm shaking. The bacterial suspension was then adjusted to a final optical density (OD_600_) of 0.05 (approximately 5 × 10^7^ CFU/ml) in 25% LB broth using a Genesys 50 spectrophotometer (Thermo Scientific, USA). A 100 µl aliquot of a normalized bacterial suspension was transferred into each well of sterile, non-treated, flat-bottom 96-well plates and supplemented with 1 µl of SMs (6.25–100 µM final concentration). Bacteria grown in 1% DMSO, chloramphenicol (20 µg/ml), and 25% media alone were used as controls. The Plates were incubated at 28 °C for 72 h in a Sunrise Tecan kinetic microplate reader (Tecan US). Plates were agitated every 15 min for 30 s at 180 rpm, OD_600_ was measured every 30 min. The cultures exhibiting complete growth inhibition (no increase in turbidity or optical density, no visible growth) were further assessed for bactericidal activity by streaking the culture onto YDC agar. The absence of colony formation confirmed a bactericidal effect. All the antimicrobial inhibition, MIC, and MBC experiments were conducted in three biological replicates.

### Time-kill kinetics

Time-kill assay was conducted according to the previously described protocol [54–56]. *Xhg* SM775-12 was cultured in 25% LB medium for 28 h at 28 °C. The log-phase bacterial culture was adjusted to an OD₆₀₀ of 0.05 (approximately 5 × 10^7^ CFU/ml) and treated with top 3 compounds (MBCs), **X2-c** (100 µM), **X12-k** (50 µM), and **X12-l** (100 µM) in sterile Eppendorf tubes with a final volume of 1 mL. The cultures were incubated at 28 °C with shaking at 180 rpm. To determine bacterial viability over time, 10 µL aliquots were collected from each sample at 0, 1, 2, 3, 4, 5, 6, 7, 8, 10, 12, and 24 h, serially diluted, and plated onto YDC agar plates. The plates were incubated at 28 °C for 48 h, after which colony-forming units (CFU) were counted to determine bacterial survival. Bacteria without compound treatment served as the control. The mean log_10_ CFU/mL of each compound at different times was plotted in the kinetic kill curve. The experiments were conducted in two independent biological replicates with two technical replicates in each assay.

### The physicochemical and ADME predictions

The smile files of compounds were generated by sketching their structures in ChemBioDraw Ultra 14. The smile files were used in the online SwissADME (https://www.swissadme.ch/) and pkCSM-pharmacokinetics (https://biosig.lab.uq.edu.au/pkcsm/) platforms to identify the physicochemical and ADME predictions of lead compounds.

## Supplementary Information

Below is the link to the electronic supplementary material.

Supplementary Material 1.

## Data Availability

No datasets were generated or analyzed during the current study.
