# Supplementary material for: Synthesis of carbazole and naphthyl-pyrimidine-based inhibitors of Xanthomonas for tomato bacterial spot management
Source: Mol Divers. 2026 Apr 4;30(4):6239–54. doi: 10.1007/s11030-026-11516-3 (PMC13333000; doi:10.1007/s11030-026-11516-3)

**SUPPORTING INFORMATION**

**Synthesis of Carbazole, and Naphthyl-Pyrimidine-Based Inhibitors of *Xanthomonas spp*. for Tomato Bacterial Spot Management**

Rajeev Shrestha,^1^ Rahul Khupse,^2^ and Gireesh Rajashekara^1^*****

^1^Department of Pathobiology, College of Veterinary Medicine, University of Illinois Urbana-Champaign, Urbana, IL 61802, USA

^2^College of Pharmacy, University of Findlay, Findlay, OH 45840, USA

**^1^H NMR and Mass Spectra of Synthesized Compounds …........................................2**

**^1^H NMR and Mass Spectra of Synthesized Compounds**

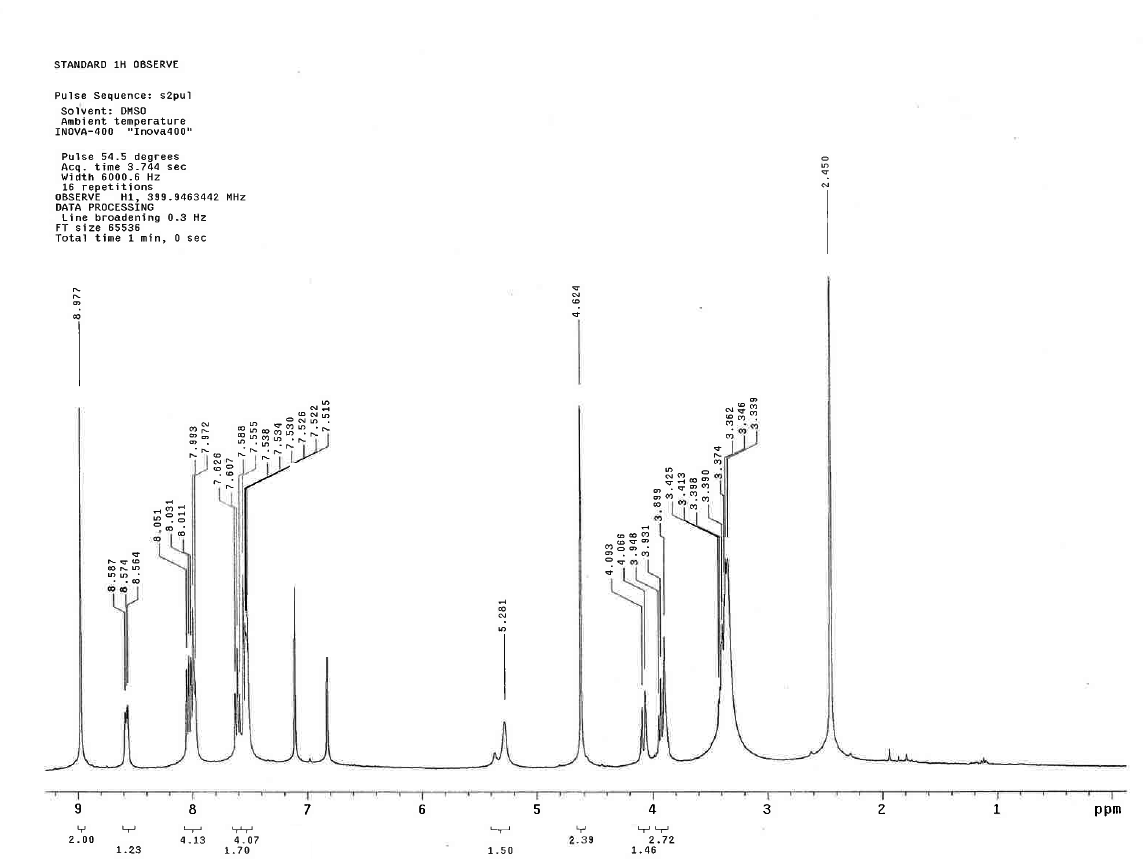

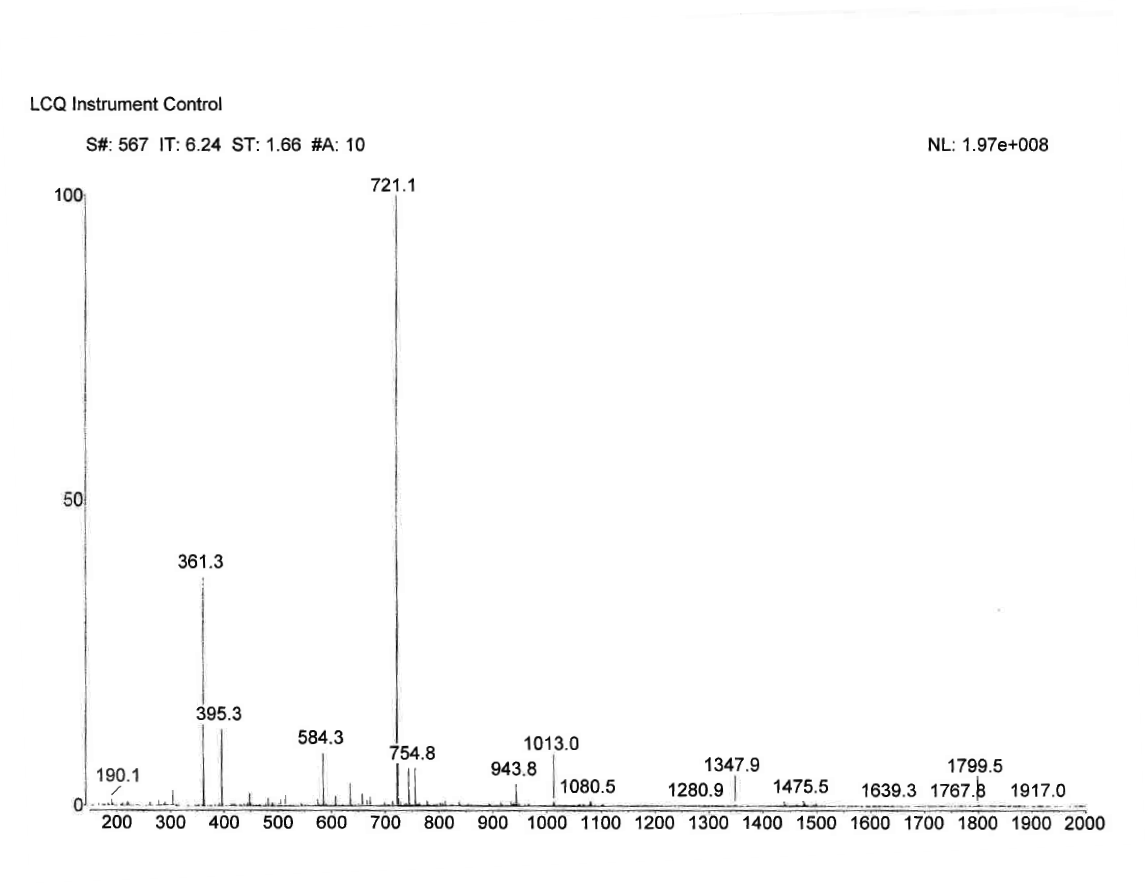

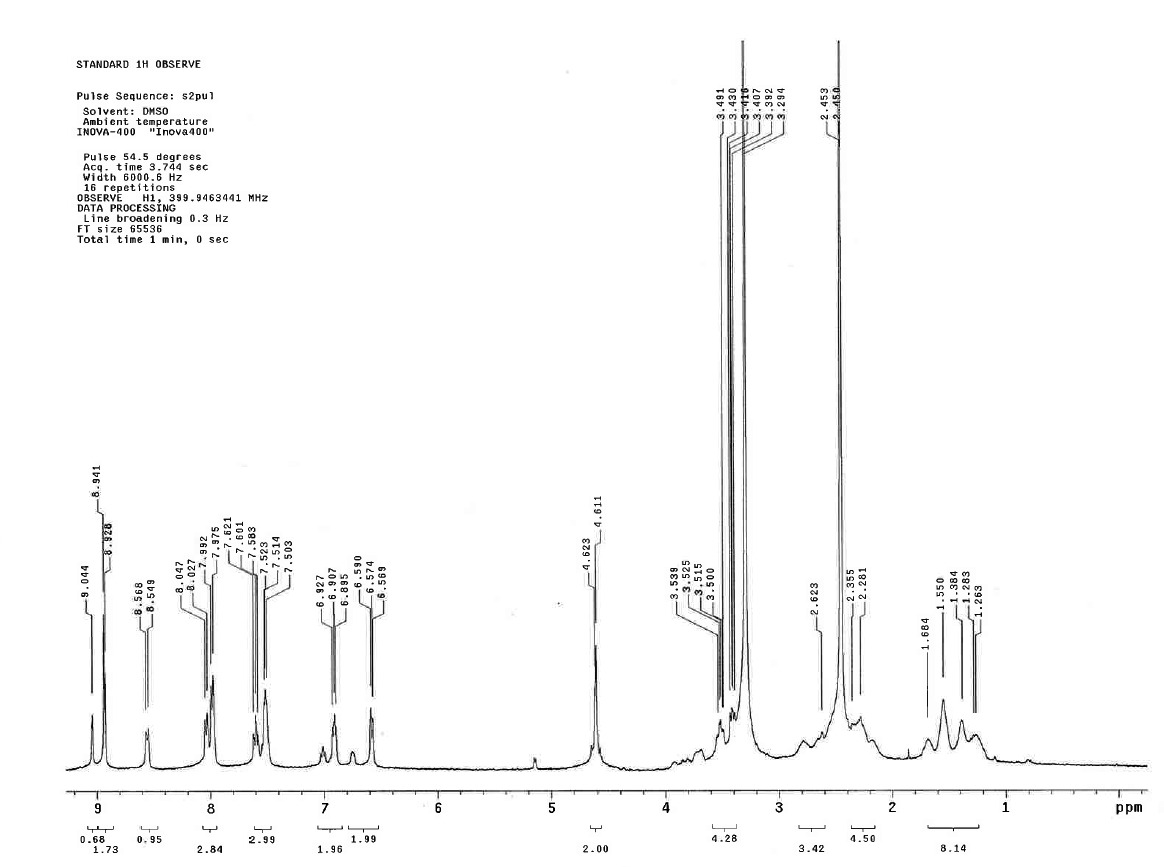

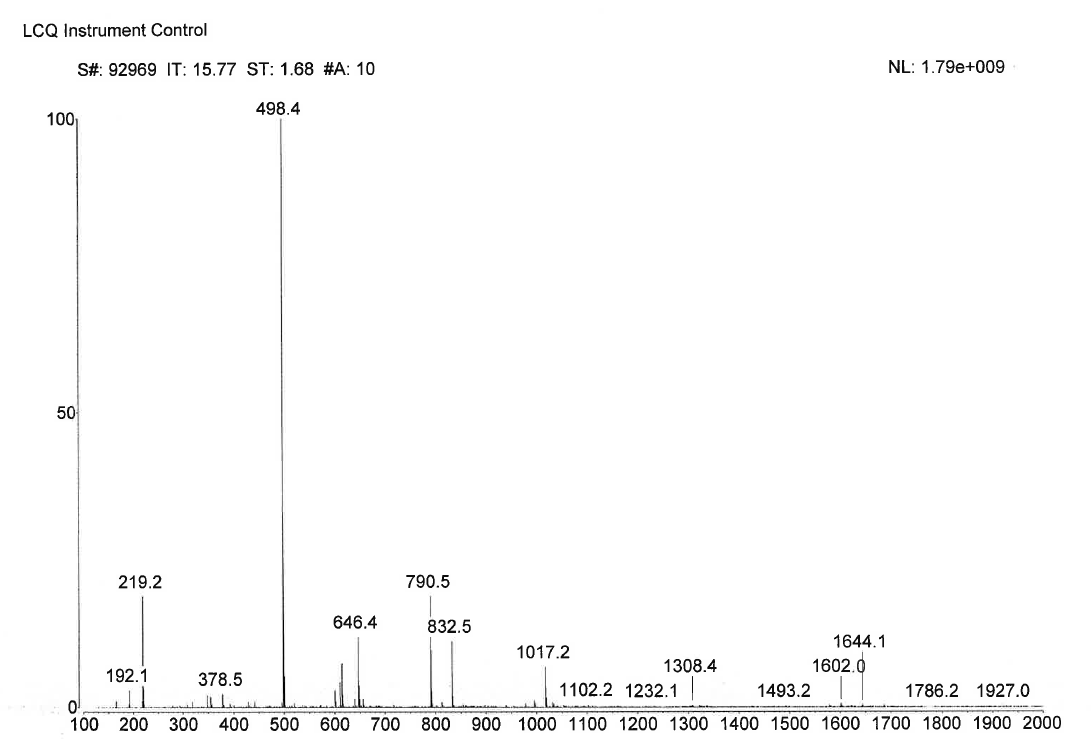


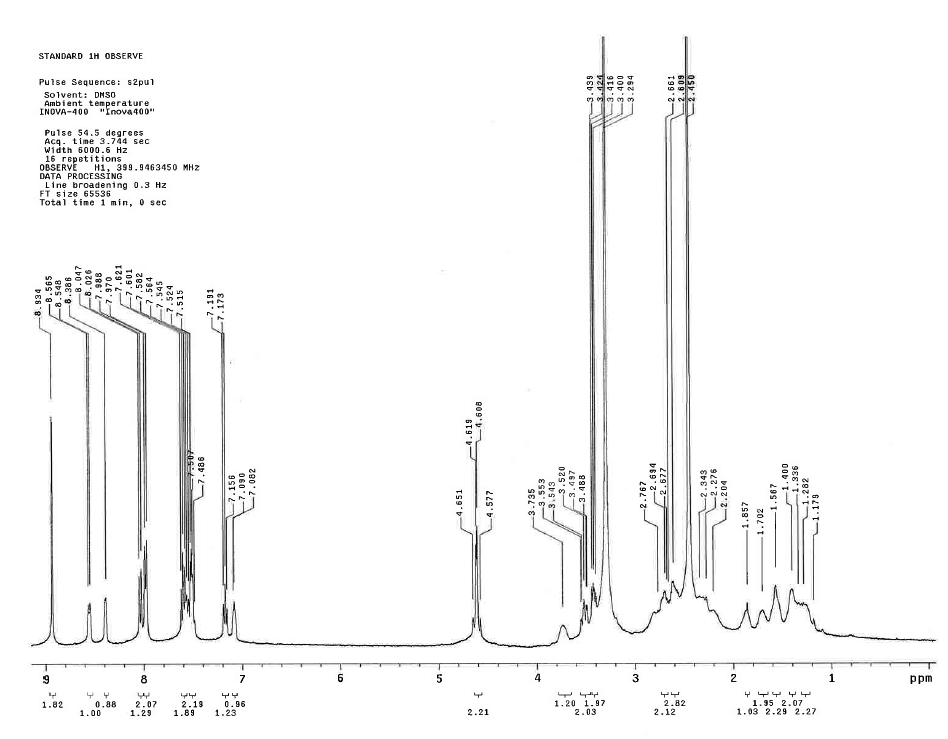

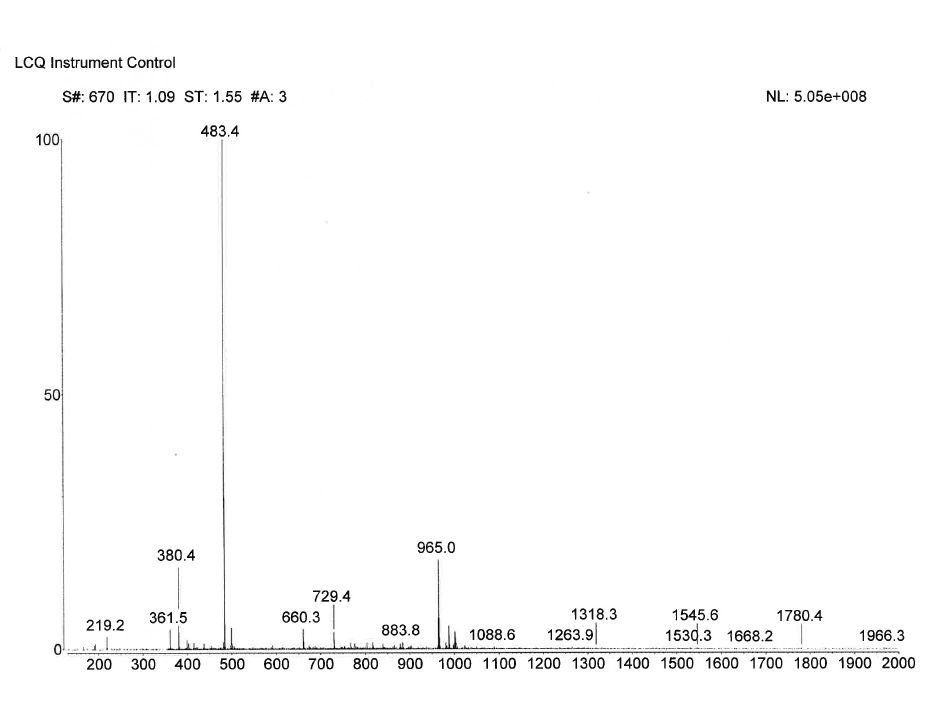

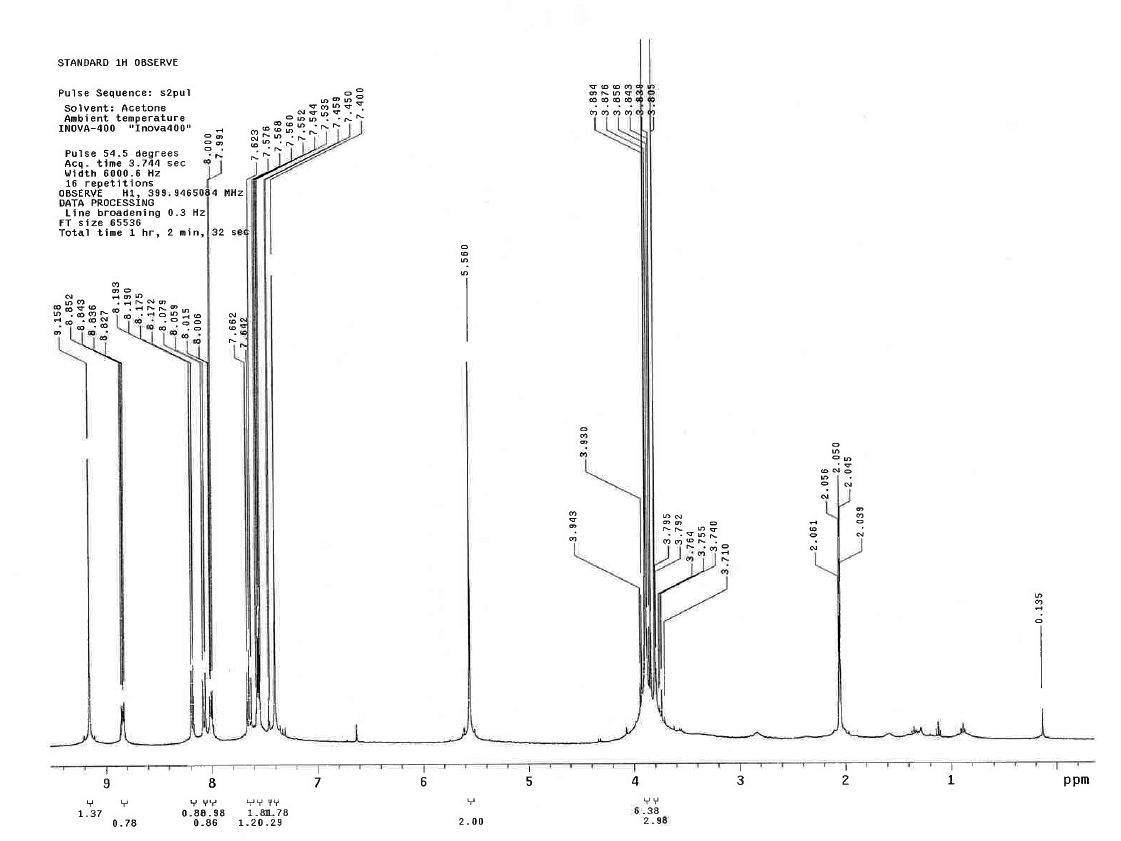

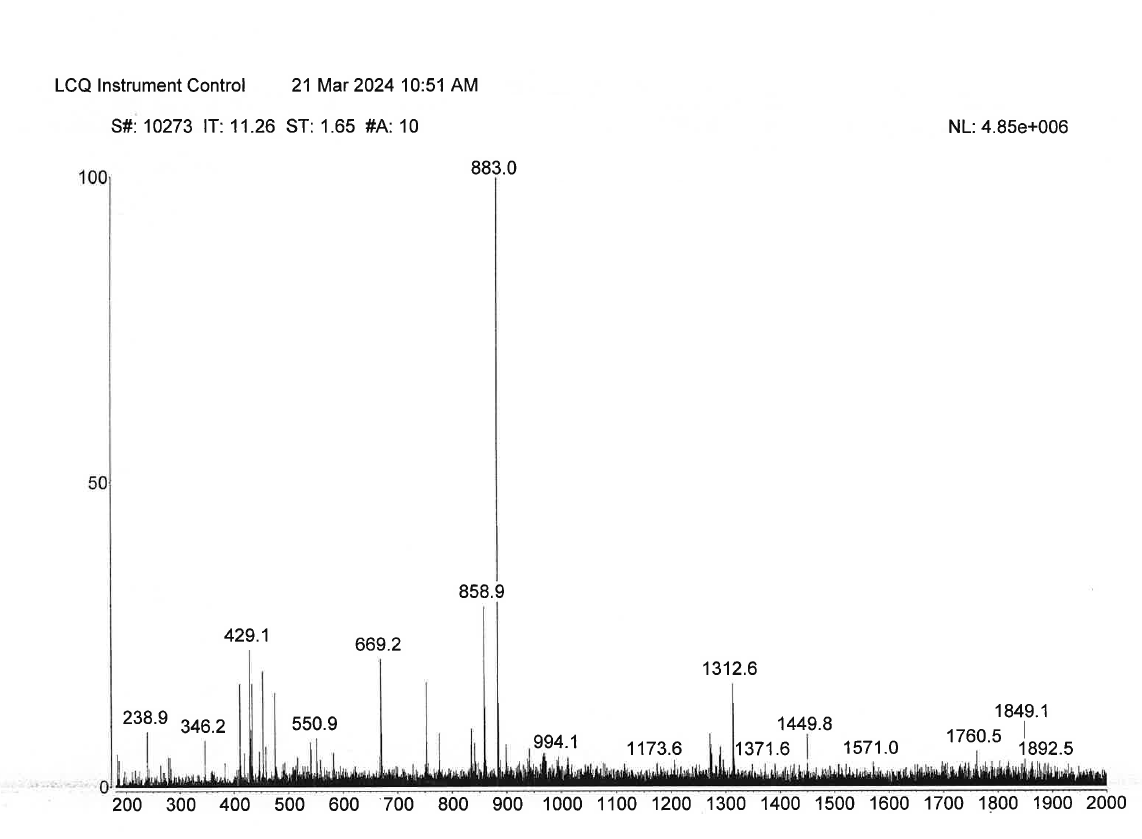

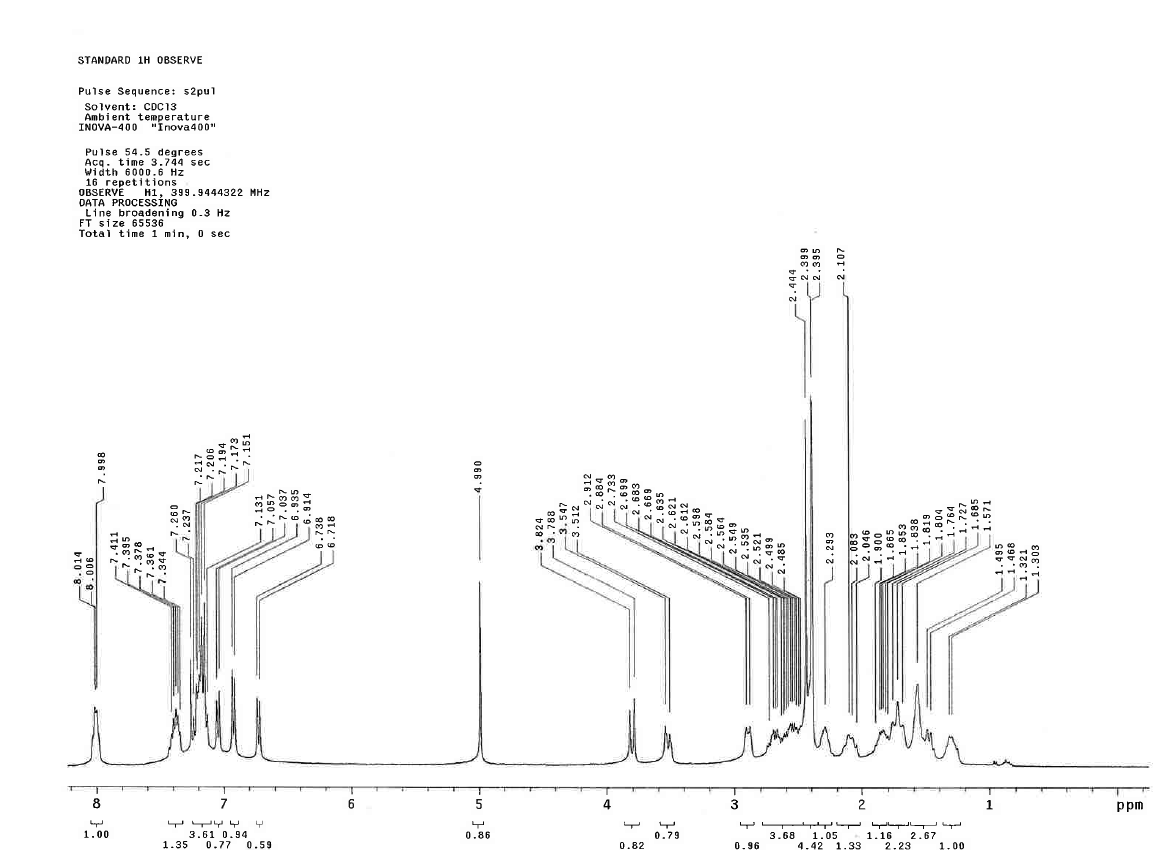

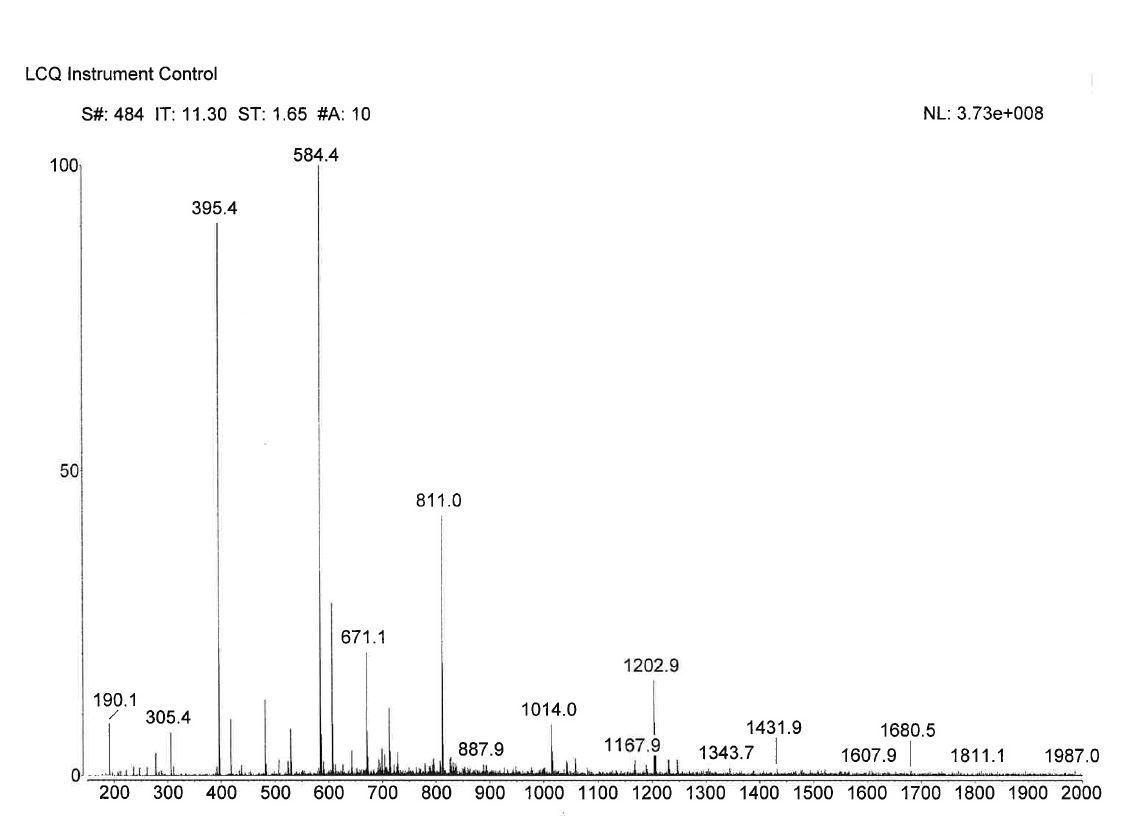

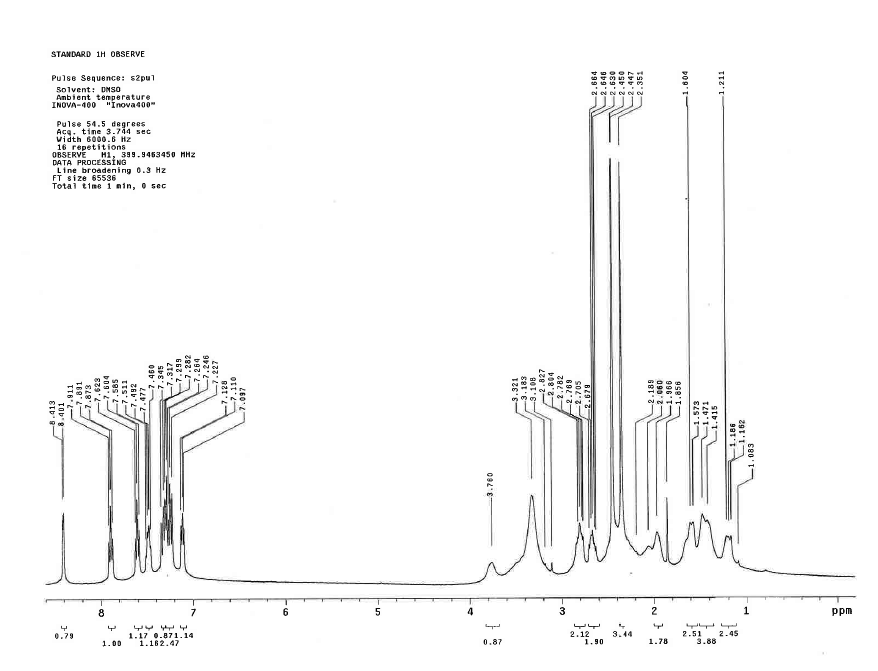

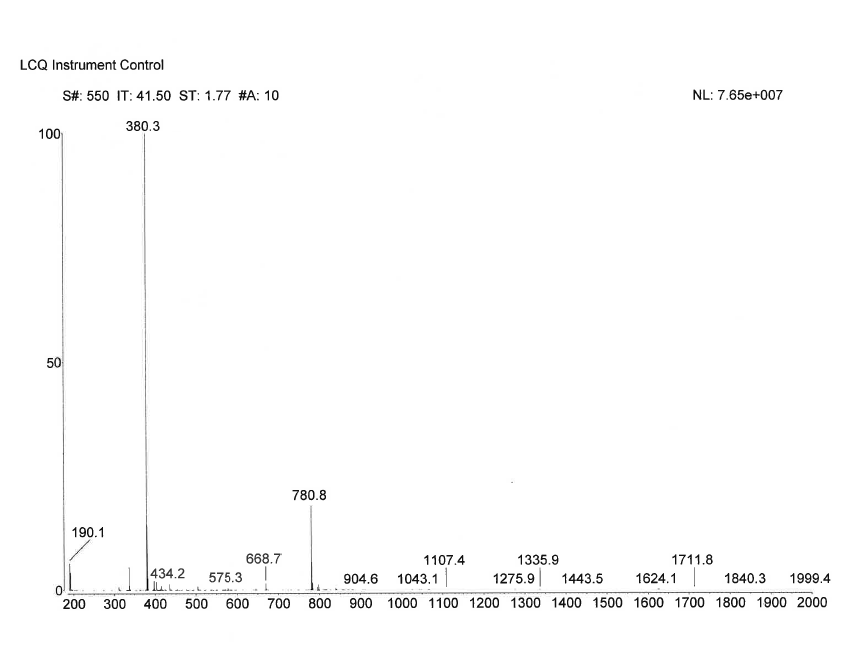

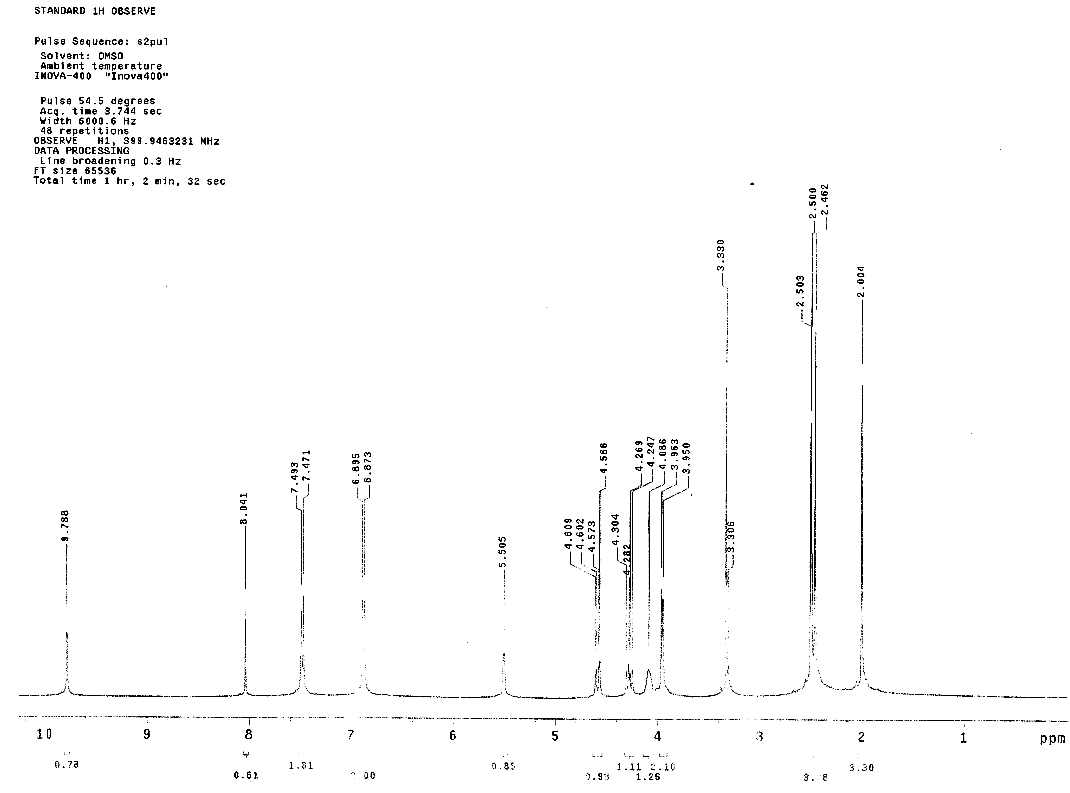


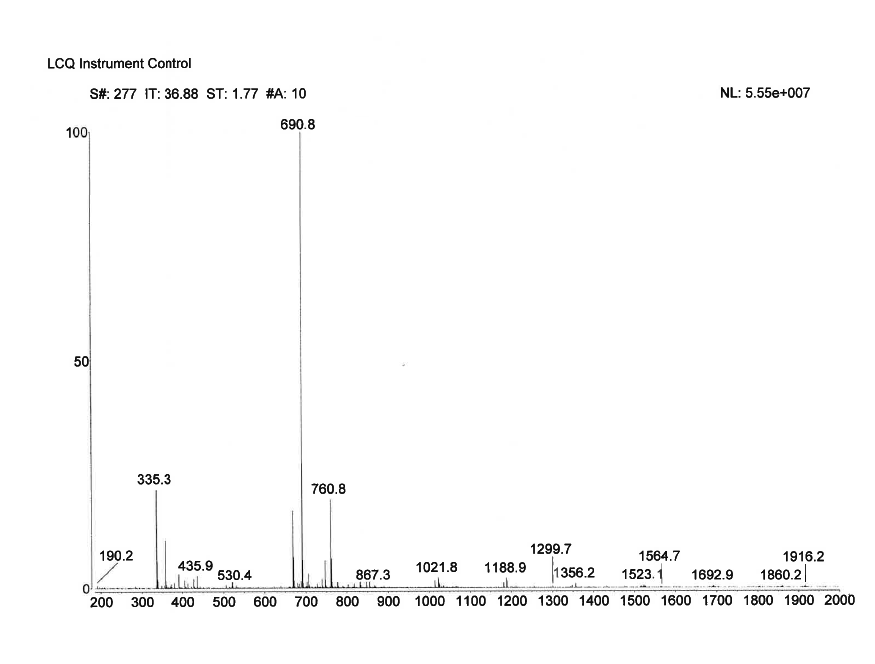

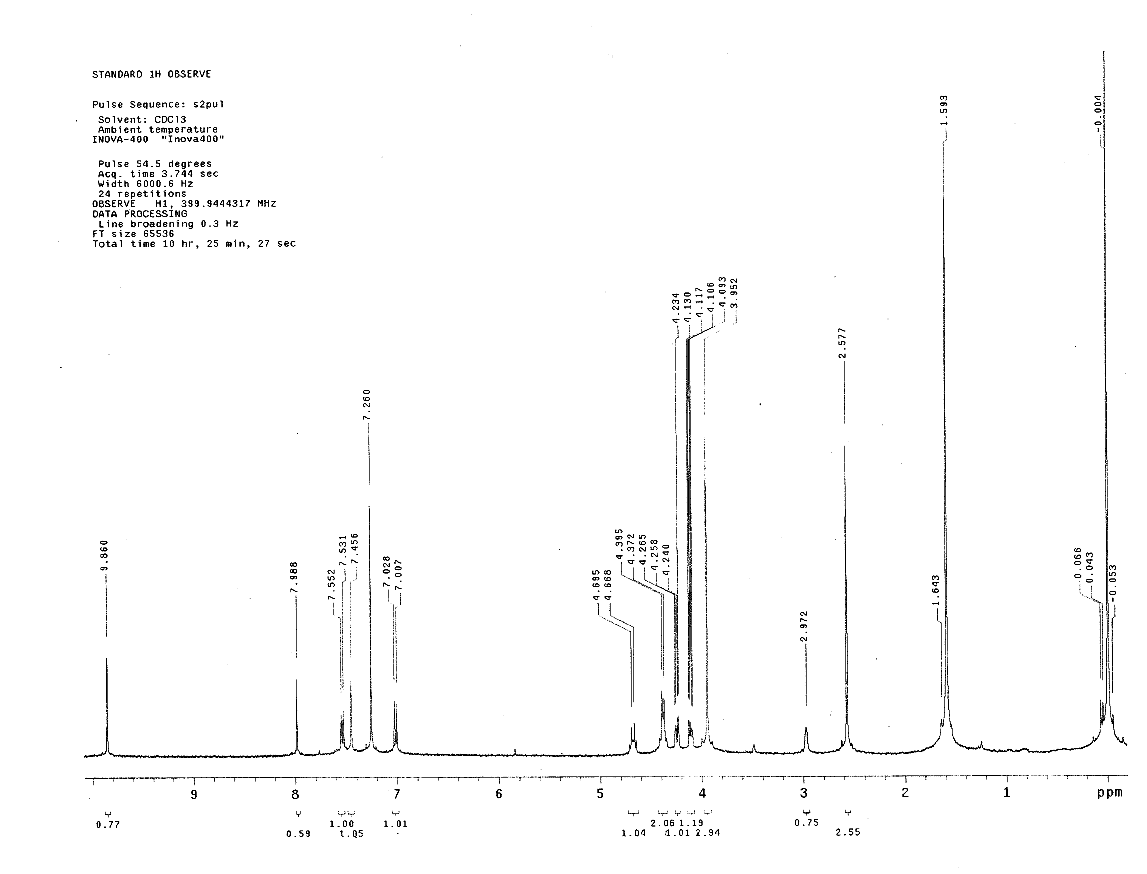


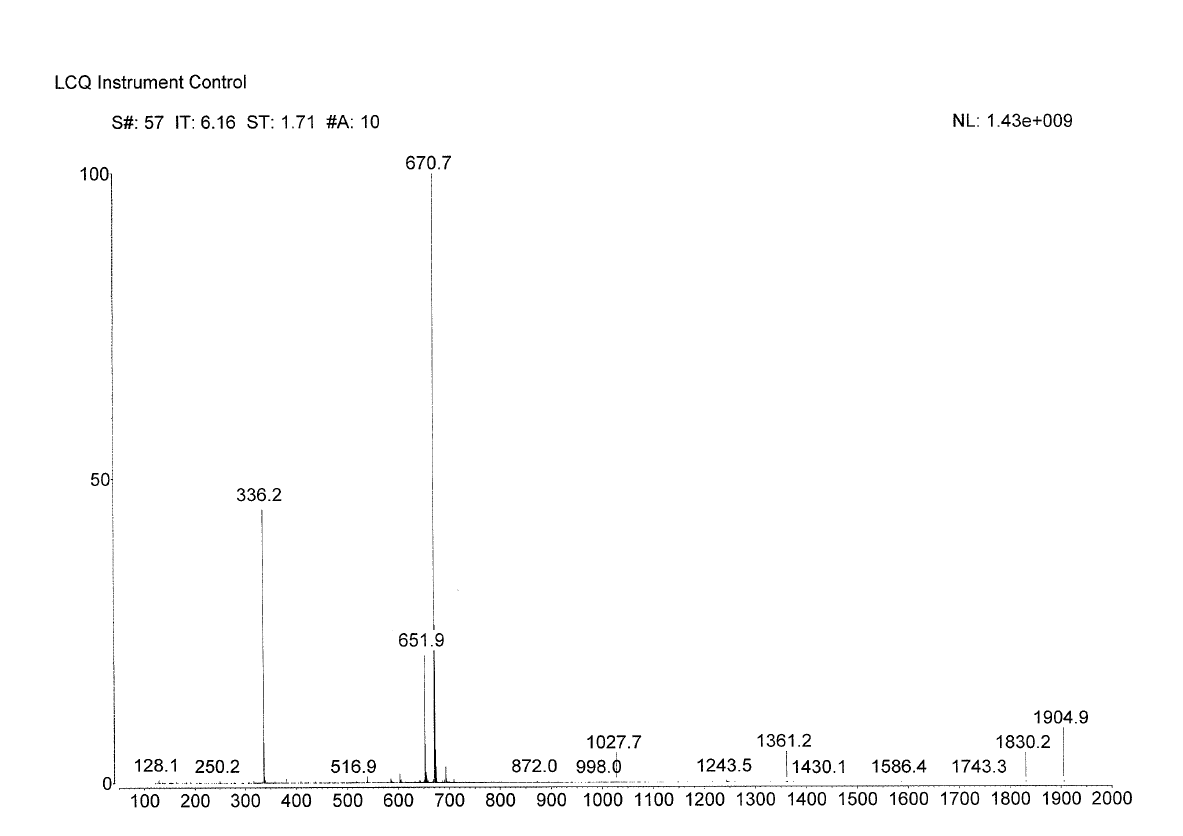

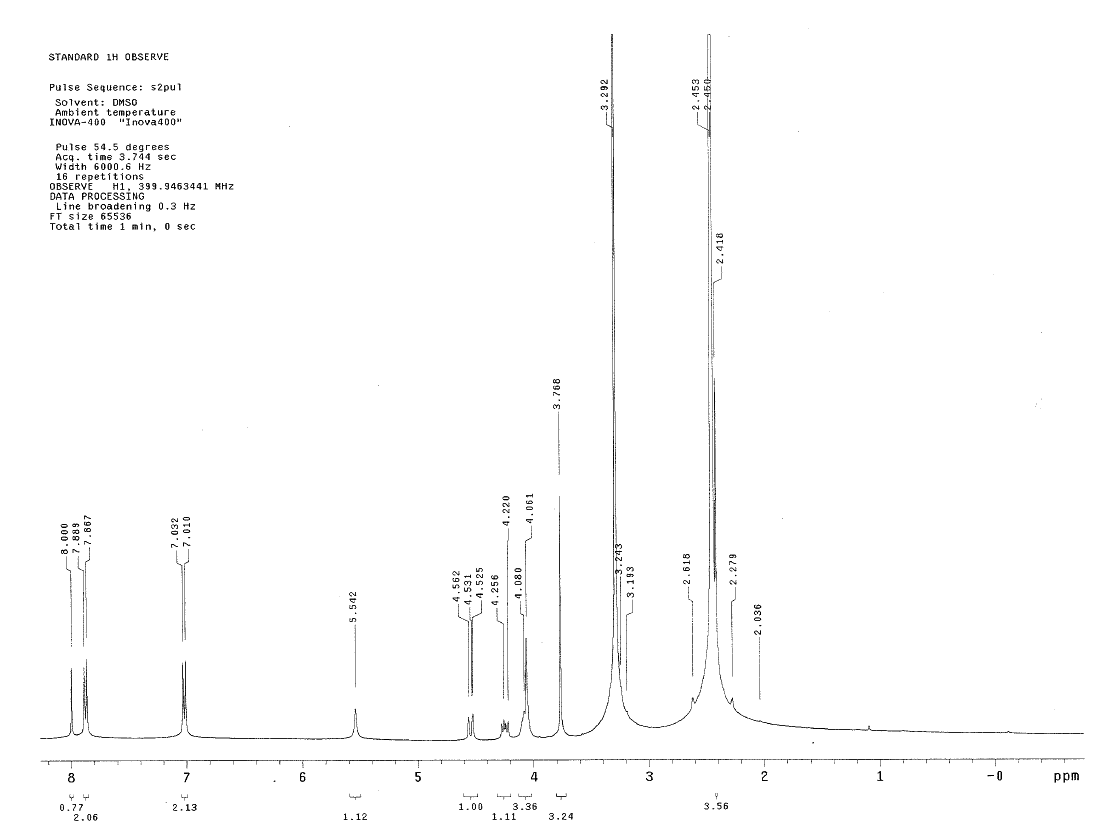

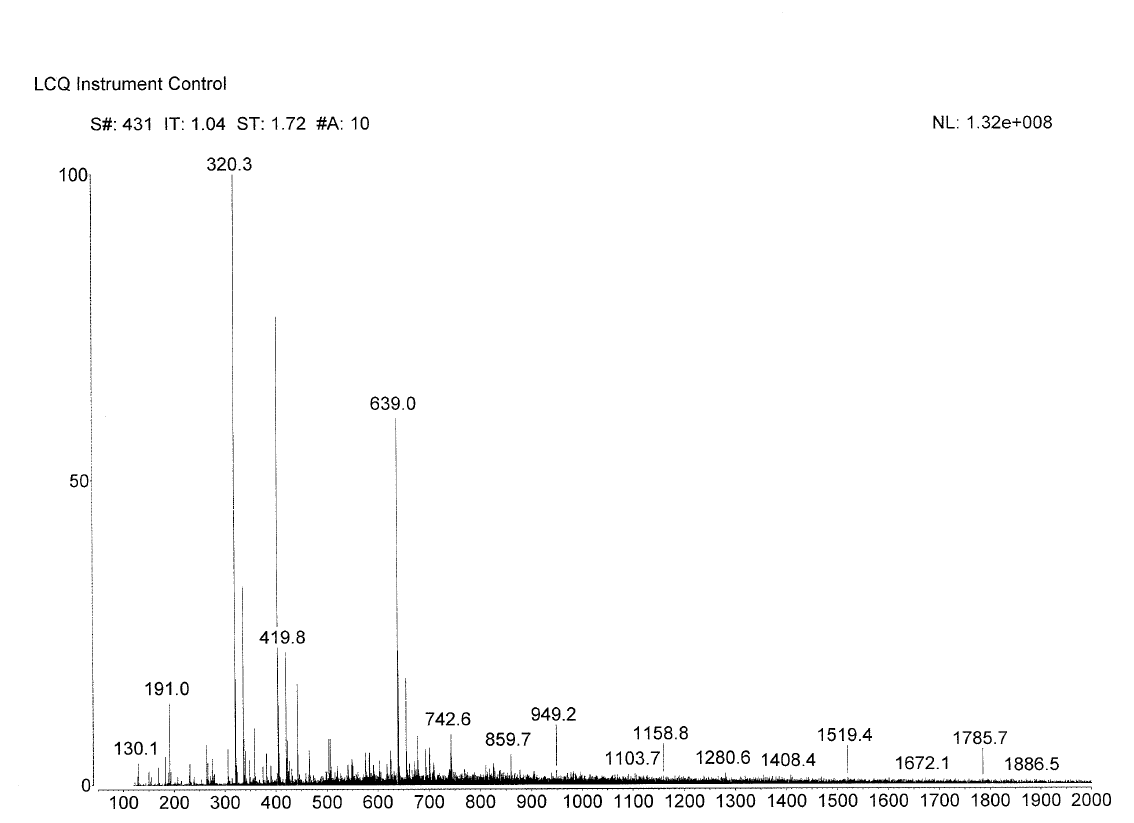

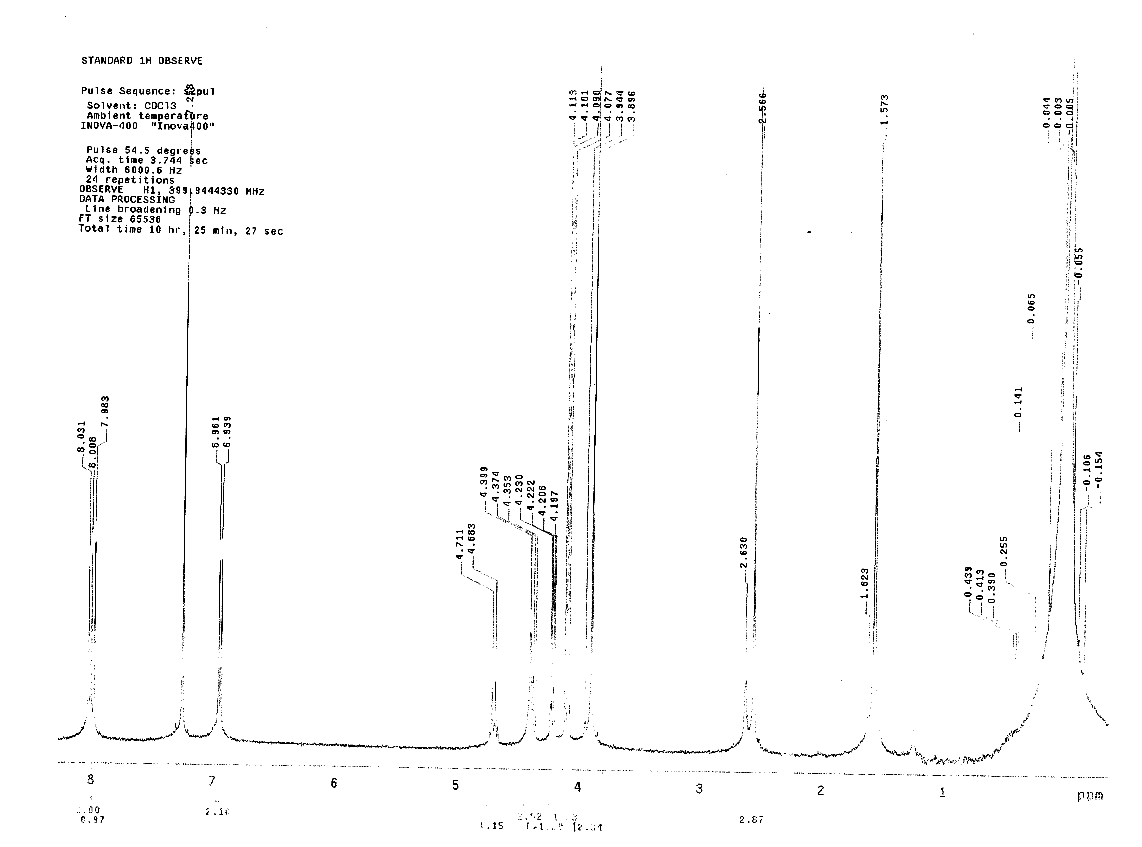

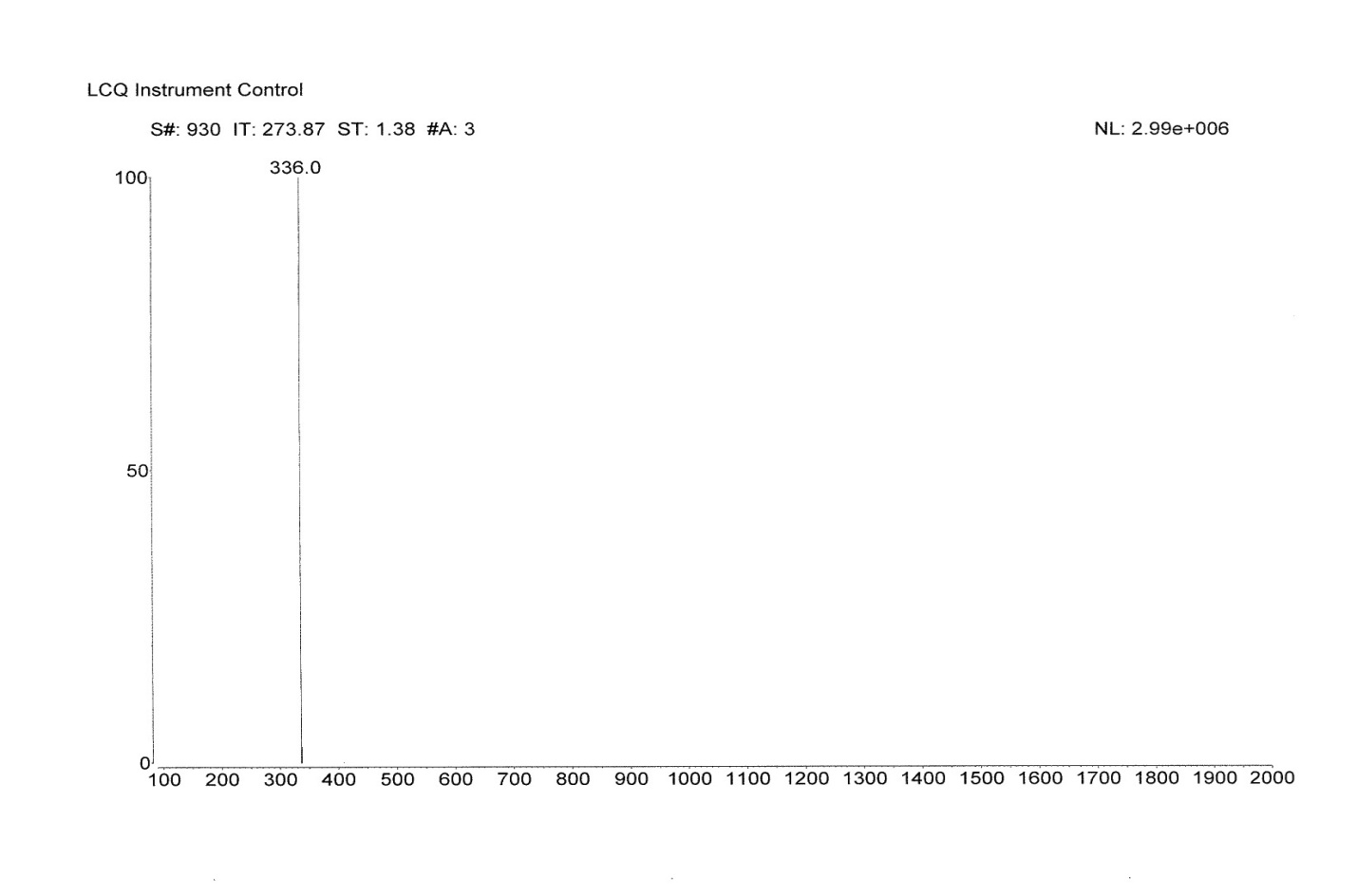

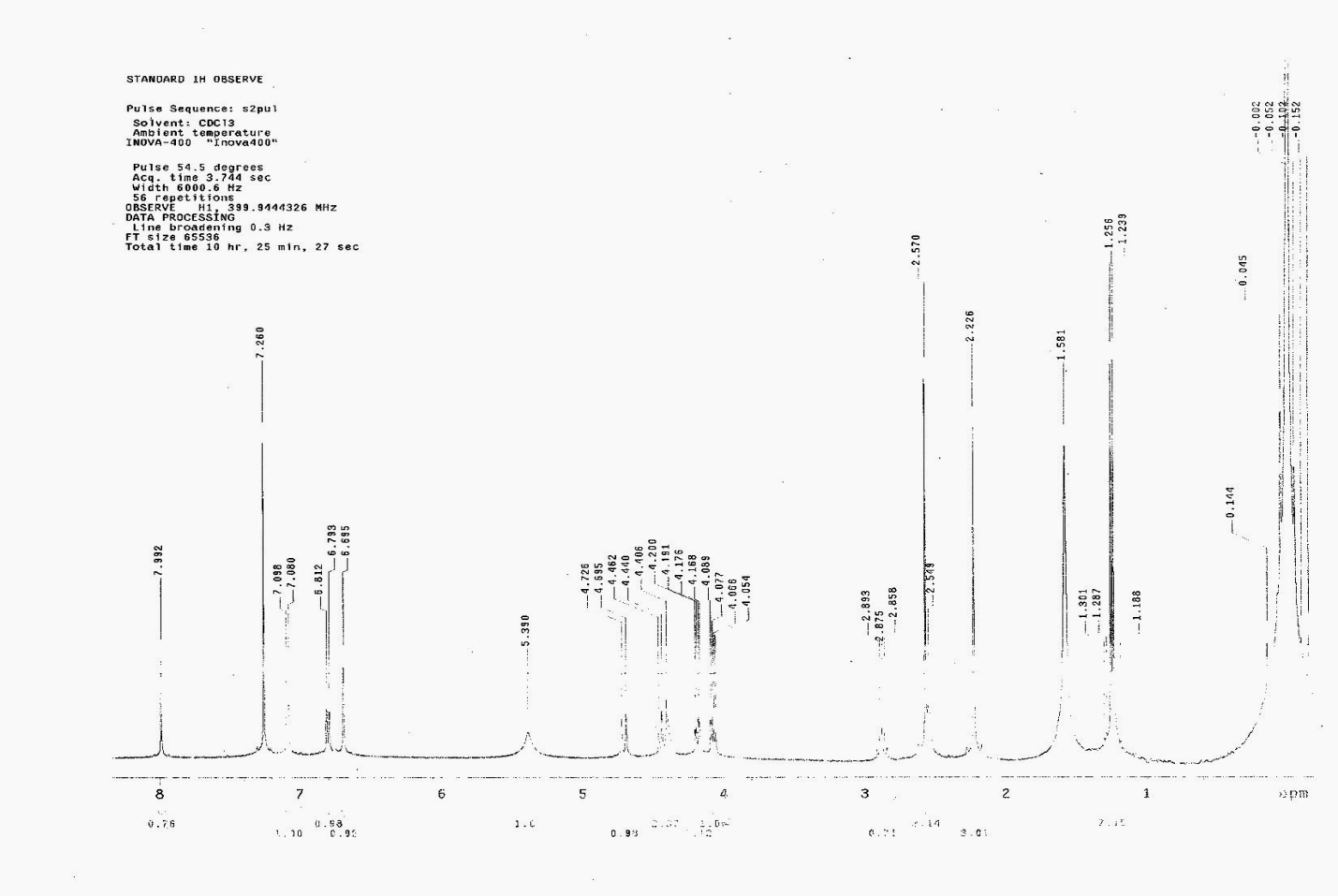

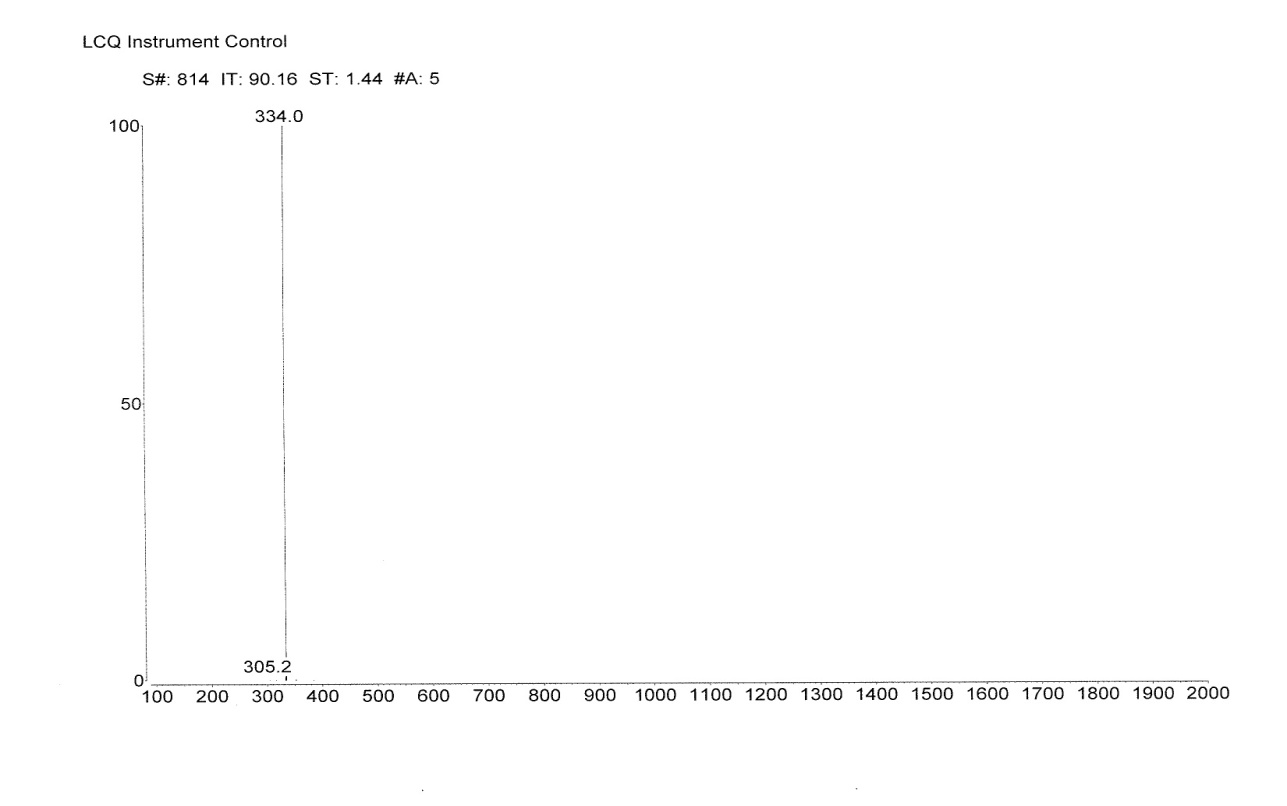

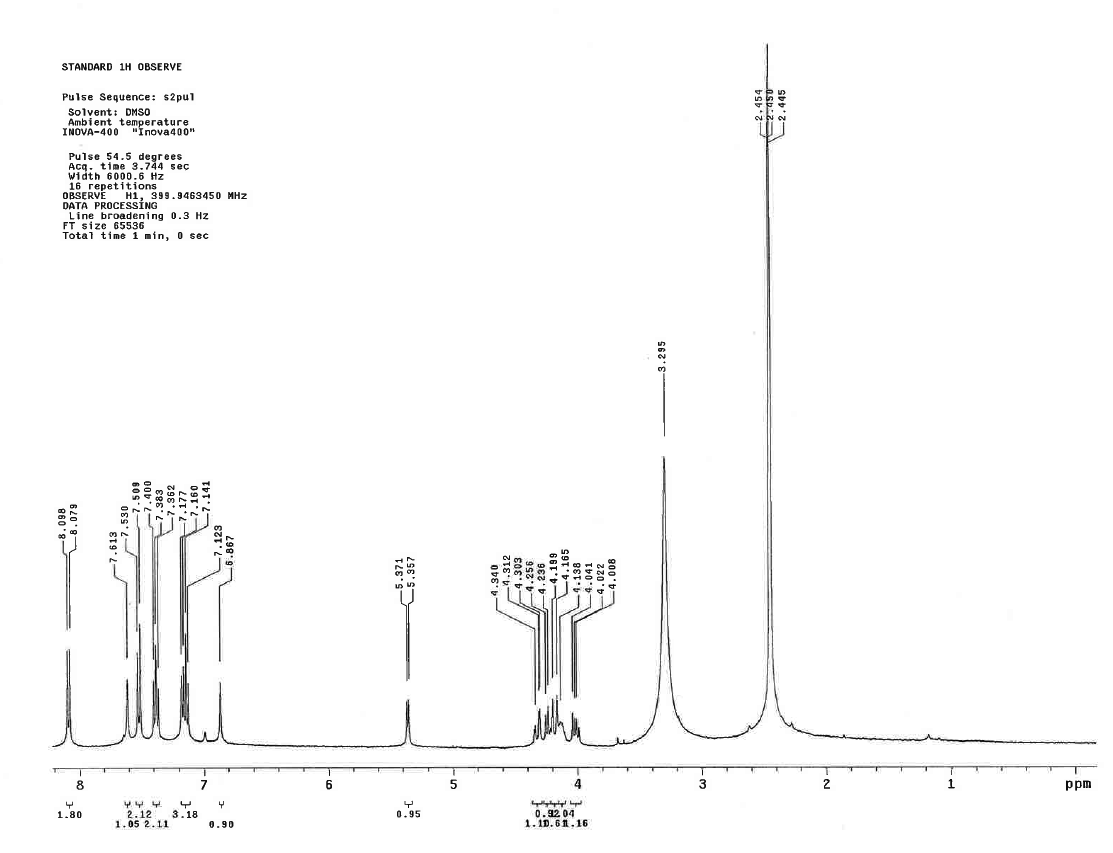

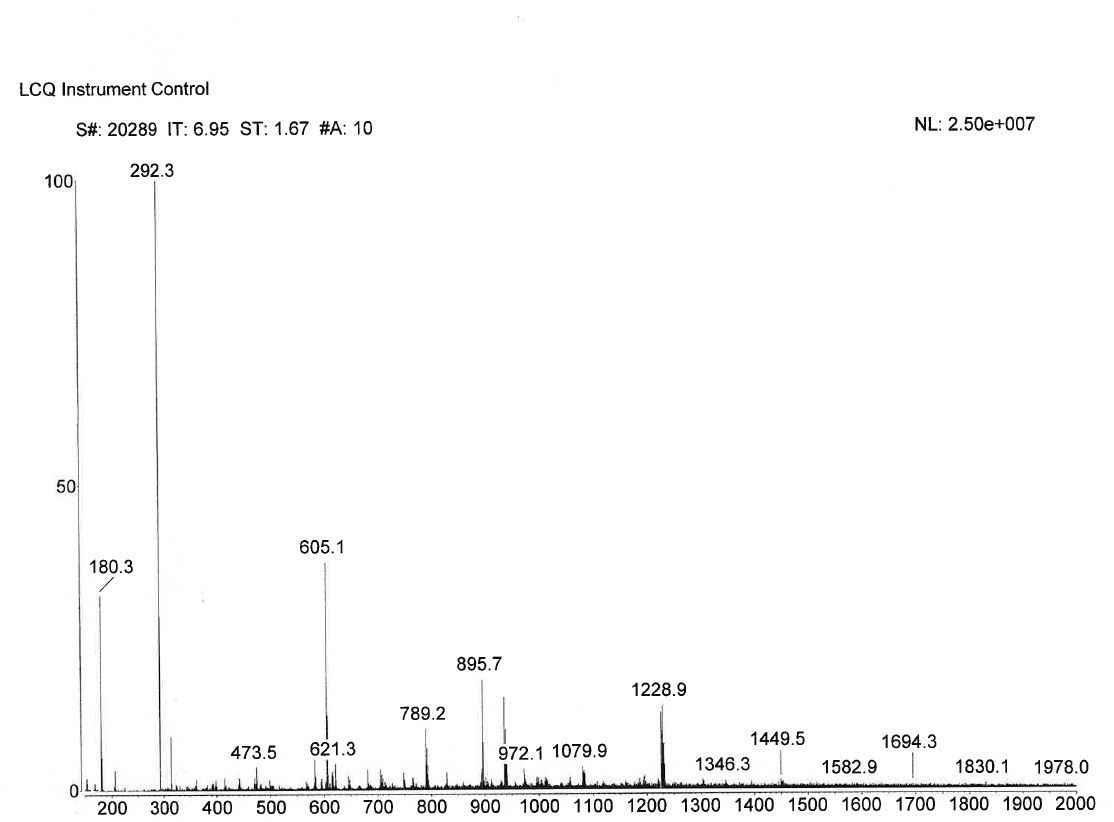

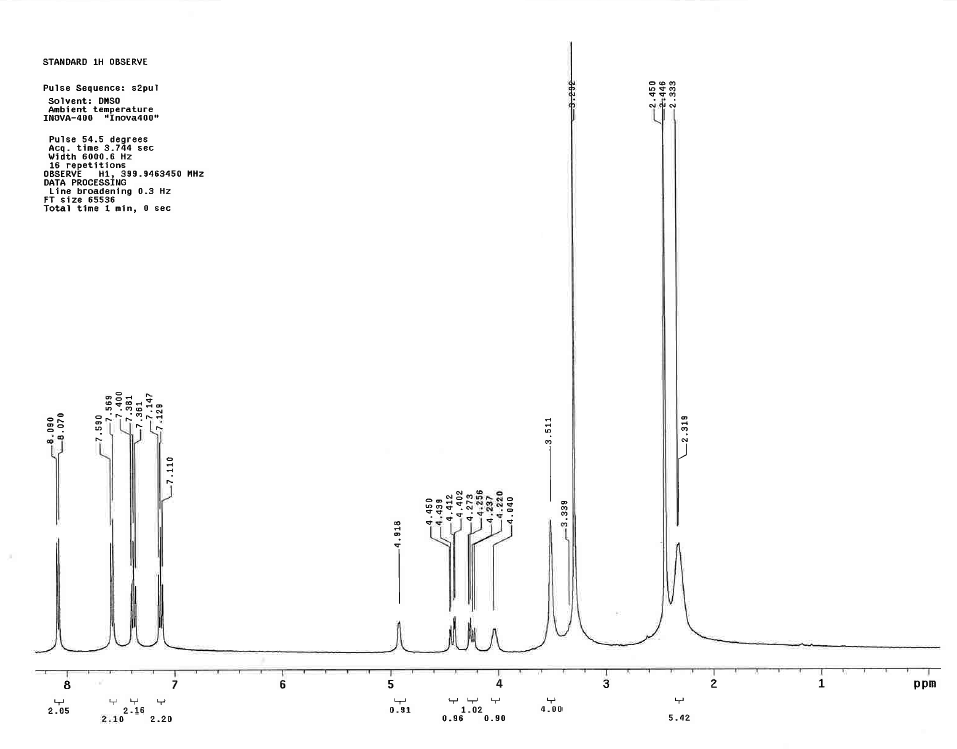

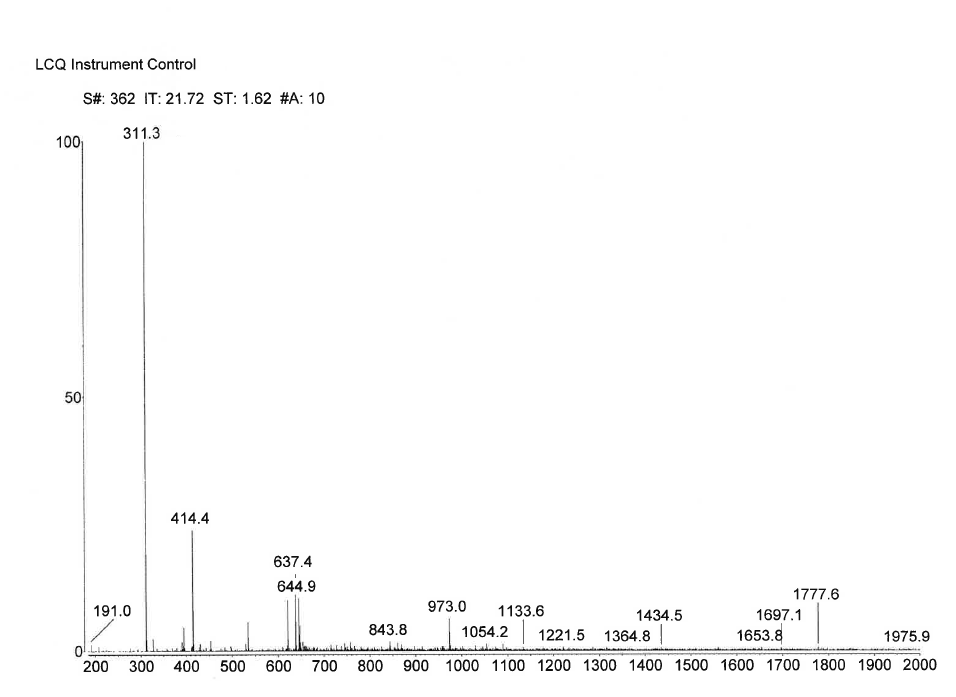

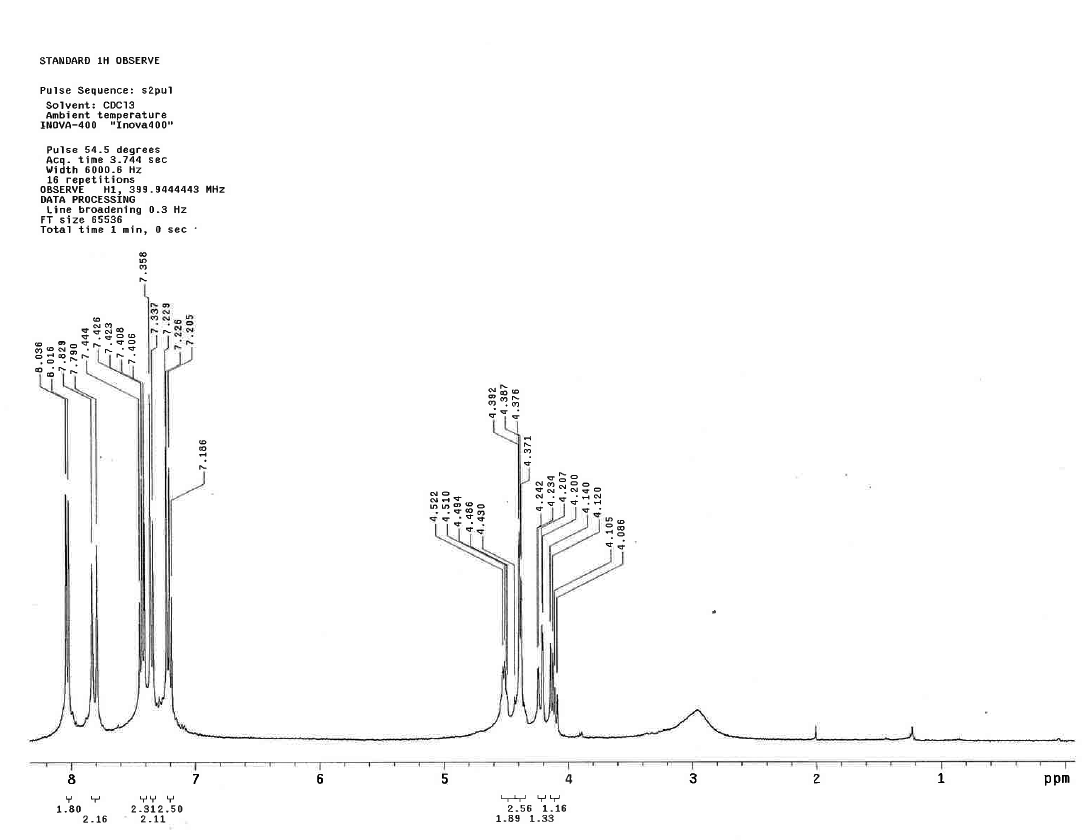


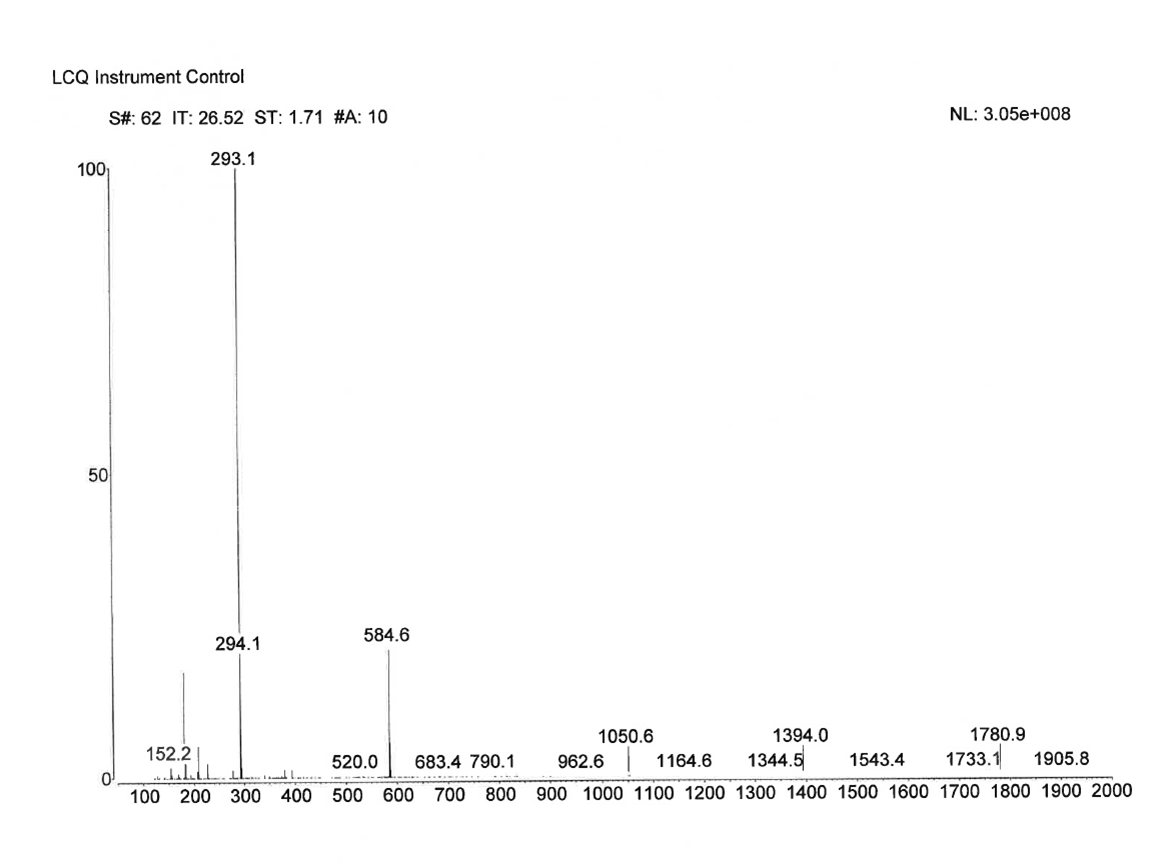

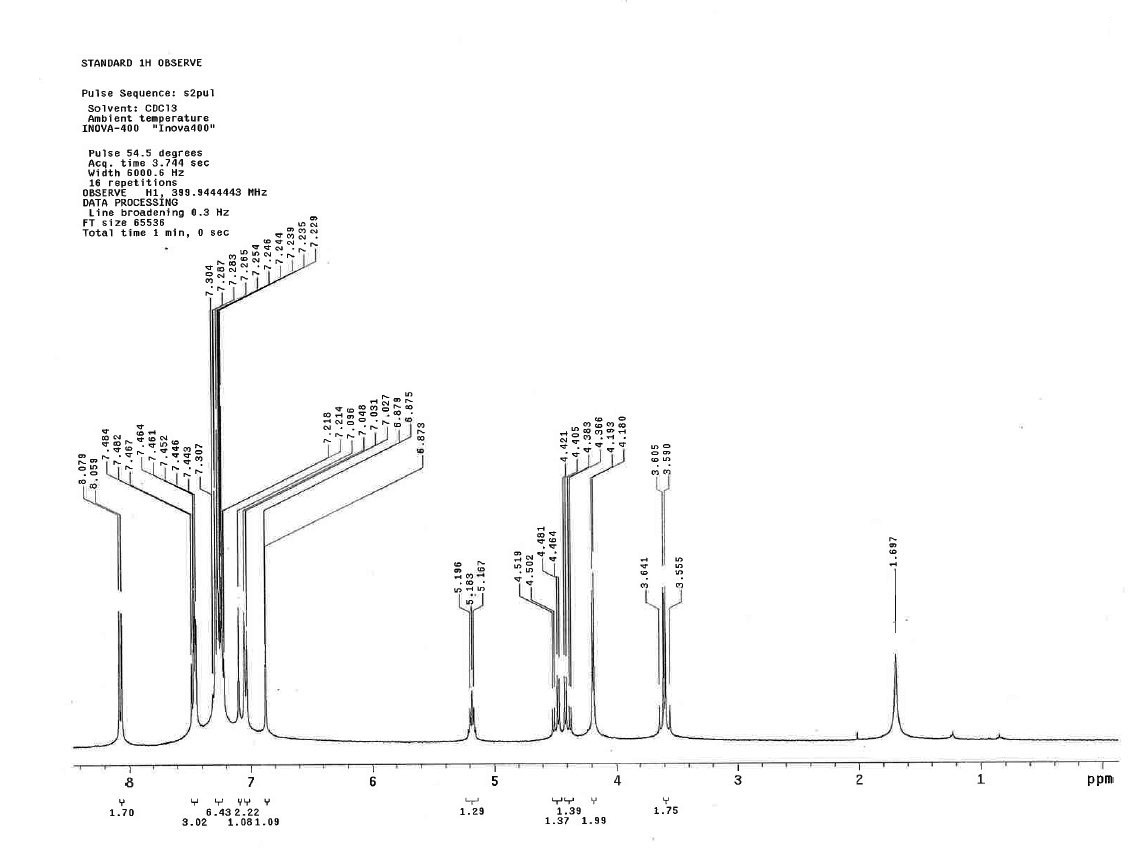


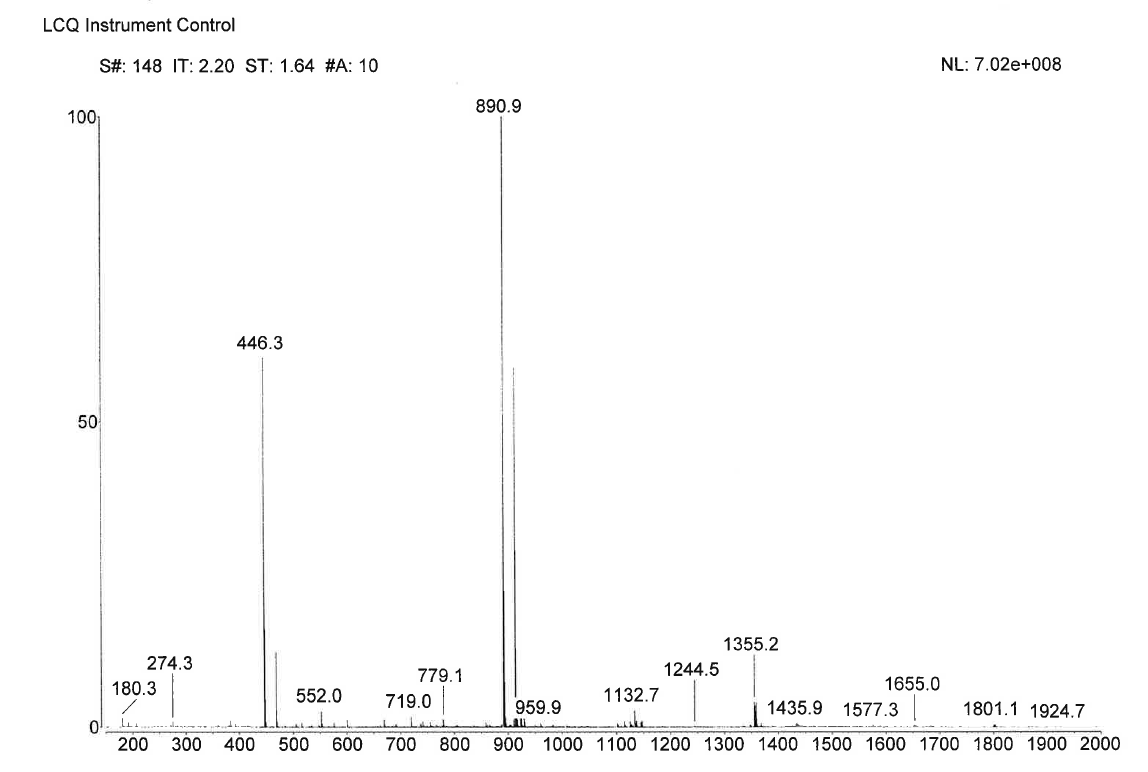

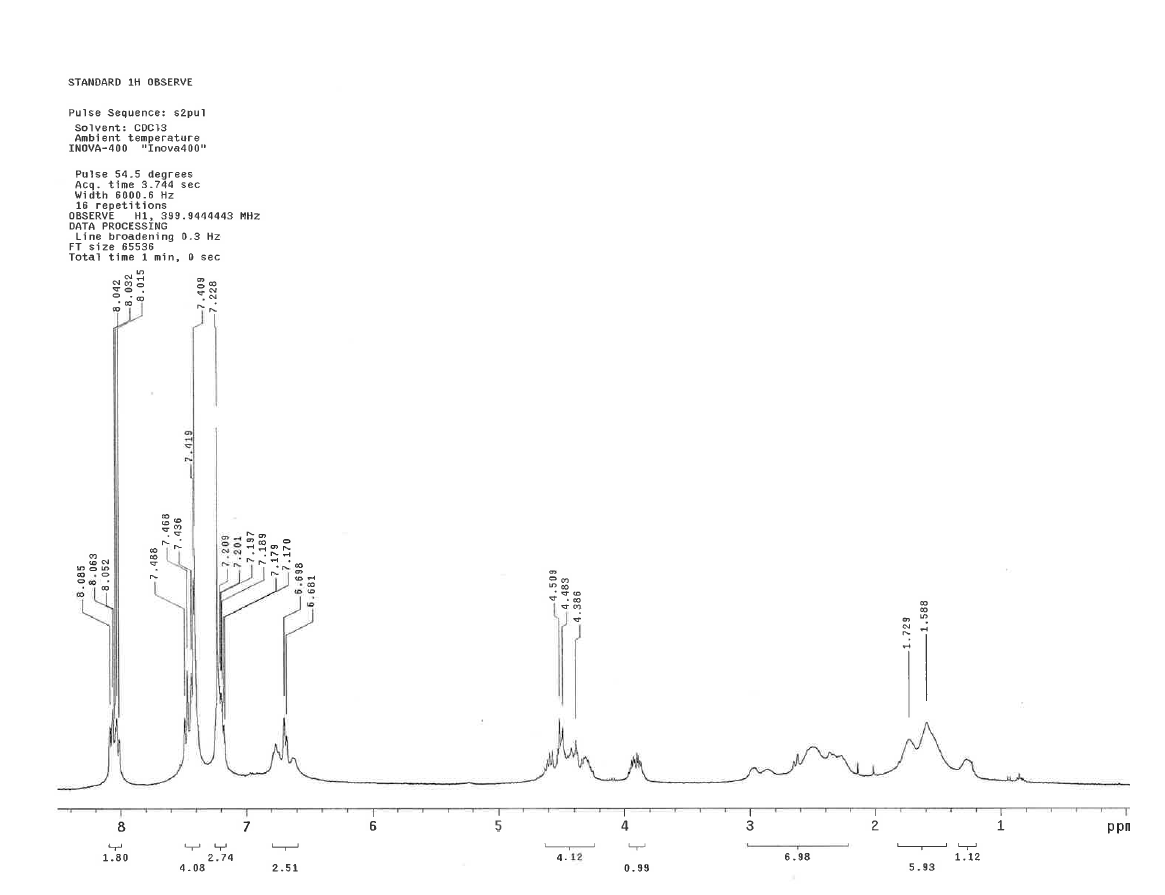


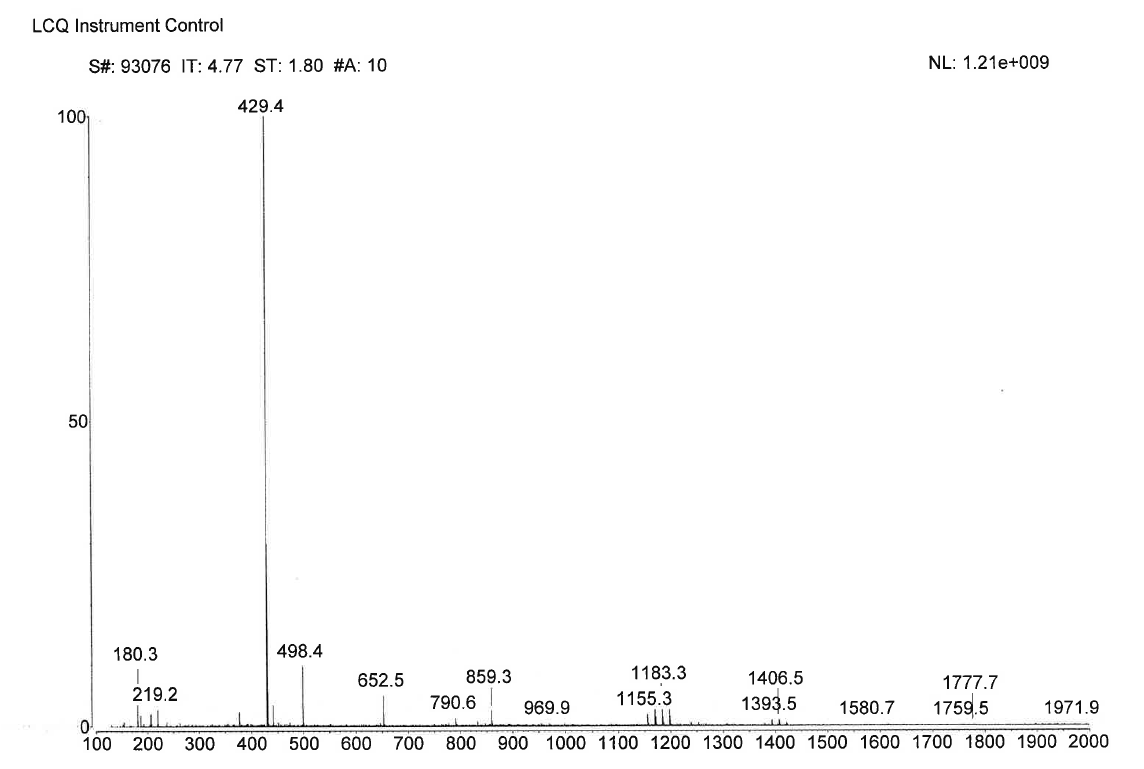

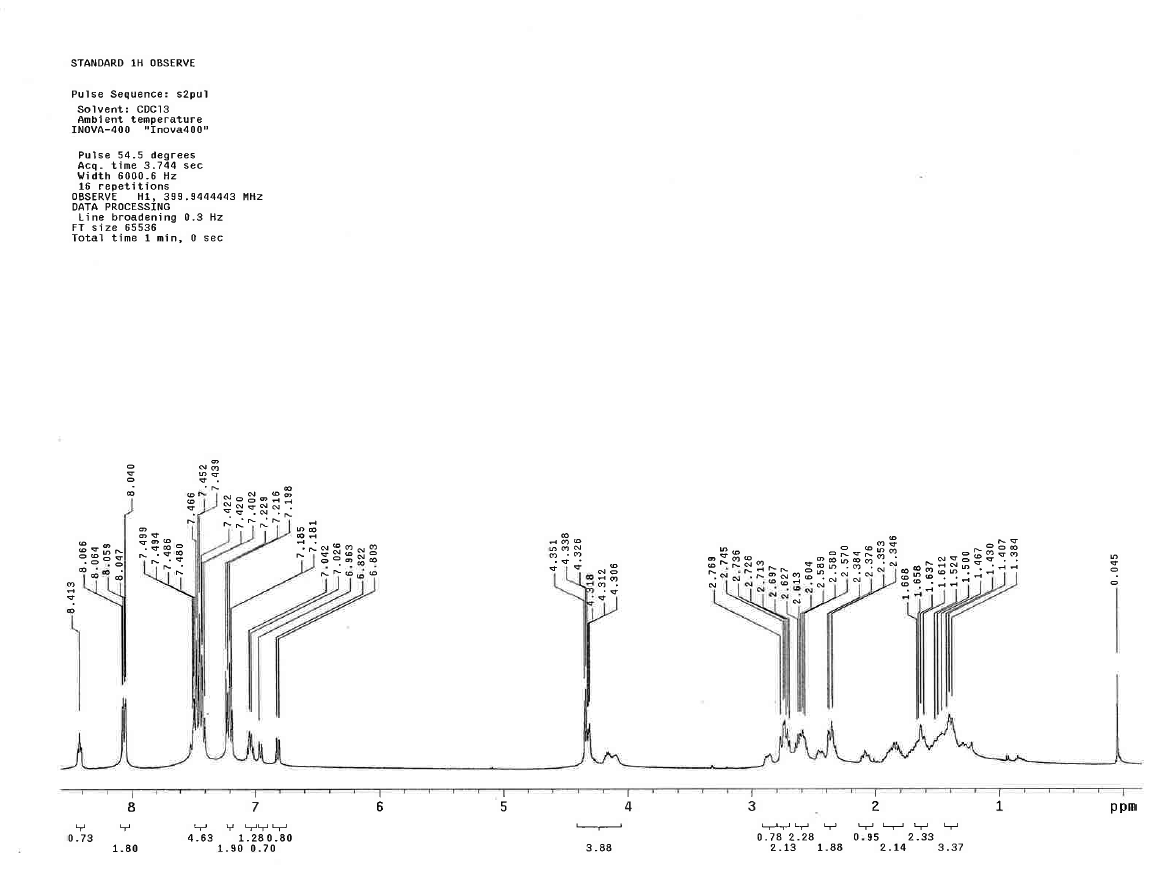

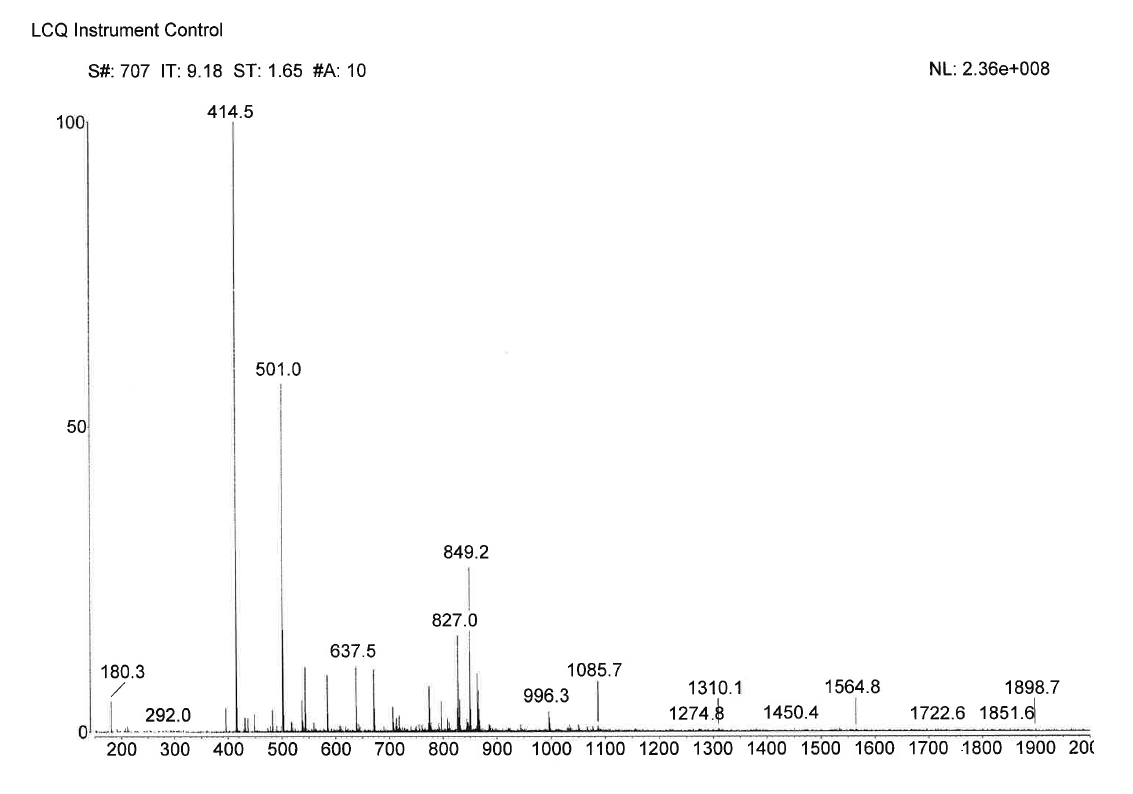

Supplement: Supplementary file 1 — Supplementary Material 1. [file 11030_2026_11516_MOESM1_ESM.docx]
